# Imaging of treatment response and minimal residual disease in multiple myeloma: state of the art WB-MRI and PET/CT

**DOI:** 10.1007/s00256-021-03841-5

**Published:** 2021-08-07

**Authors:** Frederic E. Lecouvet, Marie-Christiane Vekemans, Thomas Van Den Berghe, Koenraad Verstraete, Thomas Kirchgesner, Souad Acid, Jacques Malghem, Joris Wuts, Jens Hillengass, Vincent Vandecaveye, François Jamar, Olivier Gheysens, Bruno C. Vande Berg

**Affiliations:** 1 Radiology Department, Institut de Recherche Expérimentale et Clinique (IREC), Cliniques Universitaires Saint-Luc, UCLouvain, Hippocrate Avenue 10, 1200 Brussels, Belgium; 2 Haematology Unit, Cliniques Universitaires Saint-Luc, Institut de Recherche Expérimentale et Clinique (IREC), 1200 Brussels, Belgium; 3 Radiology Department, Universiteit Ghent, Sint-Pietersnieuwstraat 33, 9000 Gent, Belgium; 4 Department of Electronics and Informatics (ETRO), Vrije Universiteit Brussel, Avenue du Laerbeek 101, 1090 Jette, Belgium; 5 Departement of Medicine, Myeloma Unit, Park Comprehensive Cancer Center, Buffalo, NY USA; 6 Radiology Department, Katholieke Univesiteit Leuven, Oude Markt, 13, 3000 Leuven, Belgium; 7 Nuclear Medicine Department, Institut de Recherche Expérimentale et Clinique (IREC), Cliniques Universitaires Saint-Luc, 1200 Brussels, Belgium

**Keywords:** Multiple myeloma, Treatment response, MRI, PET/CT, Biomarkers, Cancer

## Abstract

Bone imaging has been intimately associated with the diagnosis and staging of multiple myeloma (MM) for more than 5 decades, as the presence of bone lesions indicates advanced disease and dictates treatment initiation. The methods used have been evolving, and the historical radiographic skeletal survey has been replaced by whole body CT, whole body MRI (WB-MRI) and [^18^F]FDG-PET/CT for the detection of bone marrow lesions and less frequent extramedullary plasmacytomas.

Beyond diagnosis, imaging methods are expected to provide the clinician with evaluation of the response to treatment. Imaging techniques are consistently challenged as treatments become more and more efficient, inducing profound response, with more subtle residual disease. WB-MRI and FDG-PET/CT are the methods of choice to address these challenges, being able to assess disease progression or response and to detect “minimal” residual disease, providing key prognostic information and guiding necessary change of treatment.

This paper provides an up-to-date overview of the WB-MRI and PET/CT techniques, their observations in responsive and progressive disease and their role and limitations in capturing minimal residual disease. It reviews trials assessing these techniques for response evaluation, points out the limited comparisons between both methods and highlights their complementarity with most recent molecular methods (next-generation flow cytometry, next-generation sequencing) to detect minimal residual disease. It underlines the important role of PET/MRI technology as a research tool to compare the effectiveness and complementarity of both methods to address the key clinical questions.

## Introduction

Multiple myeloma (MM) is the second most common adult haematologic malignancy, accounting for 10% of all haematological cancers, with an incidence of about 4/100.000/year and a median age at diagnosis of 70 years [1]. The diagnosis of MM relies on a wide range of features, including biological findings, i.e. hypercalcaemia, renal failure and anaemia, as well as on detection of lytic bone lesions on imaging studies (“the CRAB criteria”) and pathologic demonstration of bone marrow infiltration by monoclonal plasma cells or extramedullary plasmacytoma [2]. Imaging holds therefore a key position for the positive diagnosis of advanced disease as the demonstration of bone and bone marrow lesions often dictates the initiation of treatment. Modern imaging modalities have progressively replaced the historical radiographic skeletal survey (RSS) in this diagnostic role [3]. International guidelines now recommend low dose whole-body computed tomography (WB-CT), MRI covering either the axial skeleton or the whole body (WB-MRI), and 2-deoxy-8-[^18^F] fluoro-D-glucose positron emission tomography (FDG-PET/CT) as the imaging methods facing a suspected diagnosis of MM [4, 5].

These advances in imaging parallel critical improvements in treatments, with recent developments of new drug and therapeutic regimens, offering profound disease response and even a realistic hope of cure [6]. Prolonged survival with modern treatments of the disease leads to a higher proportion of patients with evolution to extramedullary or non-secretory MM cell clones which cannot be followed with haematological markers and need dedicated imaging solutions [7].

Response to therapy has been routinely monitored by quantification of serum and urine paraprotein and serum free light chains, according to the International Myeloma Working Group (IMWG) criteria which distinguishes progressive disease and stable diseases and classifies responders into complete, near complete, very good partial or partial response subcategories. Increasingly sensitive tools are needed to evaluate the deep response to current highly effective treatments and identify minimal residual disease (MRD) [8]. Among biological methods, multiparametric next-generation flow cytometry (NGF) and next-generation sequencing (NGS) can detect very subtle signs of disease within bone marrow samples.

Modern imaging methods WB-MRI and PET/CT are however progressively integrated in the guidelines and play a crucial role in the response evaluation and detection of MRD [9]. Modern biological and imaging tools should be compared and are most likely complementary tools for assessing response and MRD within and outside the skeleton and as prognostic biomarkers [10, 11]. Beside response assessment, MRI is the reference method for the evaluation of “skeletal related events” and complications of MM, allowing distinction between tumoral progression and “benign” complications of the disease and osteoporosis [12].

Here, we illustrate the imaging findings in assessment of response, using the most effective methods WB-MRI and PET/CT, review recommendations for acquiring, reading and reporting these examinations, underline key expectations from the haematologists and suggest future works focusing on imaging and biological molecular methods to optimize treatment of MM patients.

## Clinical characteristics of MM

### The disease and its treatment

MM is a heterogeneous disease that evolves from an asymptomatic pre-malignant stage termed monoclonal gammopathy of undetermined significance (MGUS). MGUS concerns over 3% of the population above the age of 50 and is associated to a progression to MM or related conditions at a rate of 1% per year [13]. The diagnosis of MM requires the presence of ≥ 10% clonal plasma cells in the bone marrow or a biopsy of a proven plasmacytoma. Symptomatic (active) MM differs from asymptomatic (indolent) MM based on symptoms related to organ damage, best known as the CRAB criteria. It also includes ultra-high-risk asymptomatic MM defined by a bone marrow clonal plasmacytosis ≥ 60%, serum free-light chain ratio > 100 and/or  > 1 focal lesion (≥ 5 mm each) detected on MRI [14].

The prognosis of MM is dependent on multiple factors, including host factors, tumour burden and biological parameters. The most pertinent host factors are age, with survival being clearly affected by the presence of frailty and comorbidities [15–17]. Tumour burden is best assessed with β2-microglobulin, the most relevant biological prognostic parameter, integrated in the International Staging System (ISS) score [18]. But the most powerful prognostic factor remains cytogenetics, since the presence of t(4;14), del(17p), del(1p) and gain 1q, as well as t(14;16) and t(14;20), is associated with a poor outcome [19, 20]. Other factors associated with aggressive disease are elevated serum lactate dehydrogenase (LDH), the presence of circulating plasma cells or extramedullary disease (EMD). All these prognostic markers have recently been summarized in the revised ISS [21].

Over the 15 last years, with the introduction of immuno-modulatory drugs (IMiDs), proteasome inhibitors and more recently anti-CD38, SLAM-F7 and BCMA monoclonal antibodies (MoAbs), major advances have revolutionized the treatment of MM. Different treatment combinations currently allow patients to achieve unprecedented rates of complete remission (CR) with extended periods free of progression and prolonged survival. Treatment of symptomatic MM relies on the eligibility for autologous stem cell transplantation (ASCT). For patients in good clinical condition, eligible for ASCT, treatment consists of 4 cycles of induction with bortezomib, thalidomide or lenalidomide and dexamethasone (VTD/VRD), followed by high-dose melphalan and ASCT and subsequent maintenance with lenalidomide. Patients not candidates for transplantation are typically treated with VRD for 8 cycles, followed by lenalidomide, or daratumumab-RD given until progression. However, despite the improvements achieved in front-line therapy, almost all patients will ultimately relapse. Treatment choices have become rather complicated in this setting and affected by various factors including the timing and aggressiveness of relapse, response and tolerance to prior therapies, age and performance status, drug availability and patient’s preferences.

### Evaluation of response and minimal residual disease (MRD)

As in many cancers, achieving complete response (CR) is the goal of treatment in MM, since it is considered the most important surrogate marker of overall survival (OS)[22]. CR is defined by a negative immunofixation and less than 5% bone marrow plasma cells. In fact, the true value of CR relies on the absence of a detectable MRD [23]. As MRD has been shown to supersede CR, it has now been incorporated in the updated response criteria of the IMWG [9].

Methods for evaluating MRD have significantly improved over recent years. NGS and NGF cytometry are now able to detect MRD at levels as low as one cell in one million of total examined cells (10^–6^). MRD detection power of these two techniques is superimposed and their use is based mostly on local availability. NGF identifies myelomatous plasma cells based on light chain clonality of phenotypically aberrant tumor cells using monoclonal antibodies combinations [24]. NGS identifies clonal immunoglobulin gene rearrangements unique to myelomatous plasma cells—while performing millions of reads of DNA fragments [25]. Both techniques have advantages and disadvantages [9]. NGS has a higher sensitivity and can detect rare residual myeloma cells within the bone marrow at the level of 10^–6^. It can be performed retrospectively on frozen samples but requires a baseline sample for the detection of the patient-specific clonal rearrangement. NGF can be done without initial sample and is more affordable [26].

As MM is a heterogeneous disease with various infiltration rates related to the patchy pattern of bone marrow involvement, other potential pitfalls have to be considered. Samples can be haemodiluted and cannot guarantee the absolute absence of clonal plasma cells. In order to avoid false MRD-negative results, a second systematic assessment is recommended [9]. In addition, bone marrow MRD does not identify extramedullary disease (EMD), a common state in later stages of the disease as well as in patients with adverse cytogenetics such as del (17p). In a recent publication, it was reported that 7% of patients with undetectable MRD progressed at a median follow-up of 40 months, with half of them having EMD at diagnosis/relapse [27]. For all these reasons, bone marrow MRD assessment has to be combined with imaging. In newly diagnosed (ND) MM, the combination of molecular and imaging techniques has a direct impact on prognosis with patients who are double negative by these 2 approaches having the best outcome [9, 28].

MRD has a prognostic value. Two meta-analyses have confirmed that achieving MRD negativity is associated with a favourable outcome in MM, with a significant reduction in the risk of progression/death (59%/43%) and significant differences in terms of progression free survival (PFS) (54 vs. 26 months) and overall survival (OS) (98 vs. 82 months) between patients in CR with undetectable vs. detectable disease [29, 30]. Patients in CR with a persistent positive MRD have the same outcome as patients in very good partial response (VGPR) or partial response (PR), underscoring the impact of undetectable MRD in CR patients in improving PFS and OS [23].

The positive clinical impact of MRD negativity in patients with high risk (HR) disease cannot be overemphasized. Adverse prognostic features can be reversed upon achieving undetectable MRD [23, 27, 31]. Patients with HR cytogenetics at diagnosis who achieve undetectable MRD after therapy not only have a better outcome compared with patients with persistent MRD, but most importantly, experience similar survival as patients with standard-risk cytogenetics who also achieved undetectable MRD [31, 32]. Of note, whereas MRD negativity at a single time point predicts better outcome, the role of sustained MRD negativity also appears cardinal to predict longer PFS and OS. Several questions remain to be answered. At present, MRD results are recommended mainly as a prognostic metric and not used in making treatment decisions. Additional trials are needed to determine if changes in treatment should be based on this MRD status.

## Radiographs

The conventional radiographic skeletal survey (RSS) has been used for decades for the detection of typical “punched-out” osteolytic lesions with absence of reactive sclerosis that are mainly observed in the axial skeleton and proximal portions of the limbs. Their observation is indicative of advanced disease and need for aggressive treatment [33].

Over years, the limitations of the RSS have been highlighted. At diagnosis, skeletal radiographs lack sensitivity as extensive loss of trabecular bone is needed before lesion become detectable, especially in the spine and pelvis [34]. Later on during treatment, signs of healing or changes in the elementary lytic lesions in response to treatment are delayed and absent by the time of response evaluation. Radiographs remain only used as first line imaging modality in symptomatic patients although in the spine, they lack specificity to distinguish fractures related to disease progression from “benign” osteoporotic fractures [35].

Hence, the development of modern imaging modalities and demonstration of their diagnostic superiority has led to the replacement of the RSS by more advanced techniques, such as low-dose whole-body CT (WB-CT), MRI and PET/CT, both at diagnosis and by the time of response evaluation [3, 36, 37].

## Computed tomography (CT)

Whole-body low-dose CT (WB-CT) outperforms conventional radiography for the detection of osteolytic lesions, detecting more lesions and detecting lesions in a large proportion of patients with negative radiographs, as demonstrated in prospective and retrospective trials [38–40]. WB-CT may have limited value to detect early focal and diffuse marrow infiltration by MM before osteolytic bone disease appears and to detect extraosseous disease except for rare cases, which requires careful analysis of soft tissues using adequate windowing [41]. Although the superior diagnostic sensitivity of both [^18^F]FDG-PET/CT and WB-MRI over WB-CT is acknowledged, WB-CT is recommended as primary diagnostic imaging method in guidelines for the initial imaging assessment of monoclonal plasma cell disorders and of MM, due to its wider availability, high sensitivity, rapidity and limited cost compared with PET and MRI [37, 42–44].

**Fig. 1:**
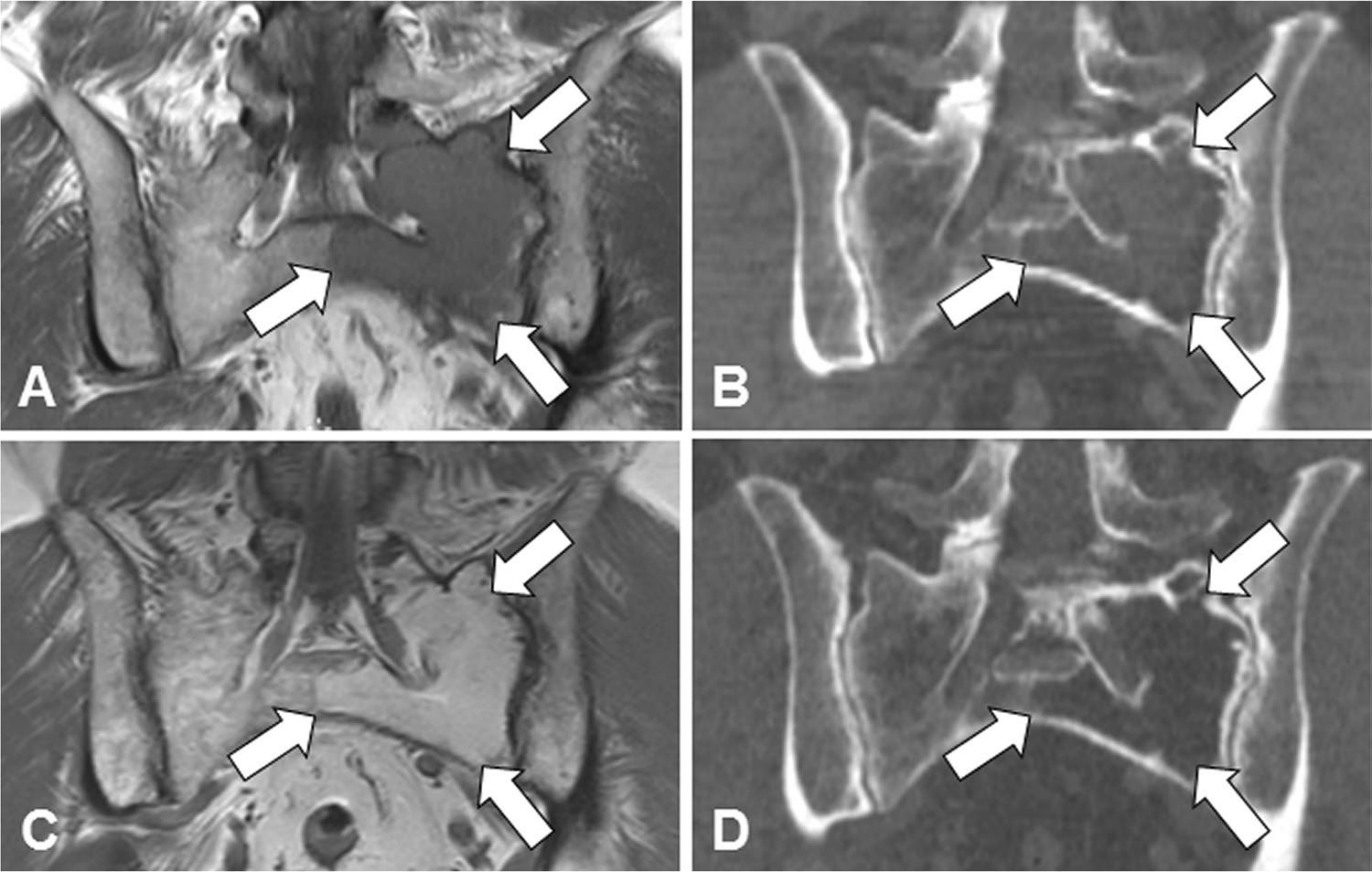
58 year-old man with ND MM (IgG kappa, stage IIIA, ISS 1) treated with high dose cytotoxic treatment and ASCT: value of morphological MRI and limited value of CT for response assessment. A Coronal T1-weigthed image of the sacrum shows extensive bone marrow lesion with low signal intensity within the left portion of the sacrum (arrows). B Corresponding reformatted coronal CT slice shows lytic lesion within the left portion of the sacrum (arrows). C 6-month follow-up coronal T1-weighted image shows disappearance of the left sacral lesion, with complete fatty replacement (arrows); no residual disease was found in the axial skeleton. D 6-month follow-up CT shows no significant change compared to the baseline: persistence of a large lytic lesion within the left sacrum (arrows).

Contrasting with its diagnostic sensitivity, WB-CT has limited interest for response assessment in MM. Indeed, in the vast majority of treated patients, WB-CT cannot discriminate between active and responsive lesions, as lytic foci often keep the same appearance for months or even years after treatment initiation (Fig. 1). CT cannot distinguish stable from responsive disease, and only lately detects progression of osteolytic lesions. Only extraosseous spread of focal bone lesions and rare extramedullary locations can be assessed for response evaluation using CT. An additional difficulty of CT results from the almost systematic use of anti-osteoclastic treatments (bisphosphonates, denosumab), which interfere with the course of bone destruction [7].

Research protocols have tried to develop quantitative tools to measure bone marrow attenuation on consecutive CTs, which should increase in stable/responsive patients compared with those with progressive disease [45]. The method remains of limited interest: in a series of 79 MM patients treated with bortezomid, only 14 showed signs of bone sclerosis on multi-detector WB-CT at 8 ± 7 months after treatment, and this sclerosis was also observed in patients with suboptimal haematological response [46]. Further studies are designed to optimize the role of CT for response assessment, relying on dual energy CT providing “virtual non-calcium” images and on the search for radiomics features correlating with haematological criteria of response [47].

WB-CT is currently not recommended for response assessment in MM [41, 48]. This inability of WB-CT to evaluate treatment response encourages the use of more sophisticated WB-MRI and PET-CT in this indication. This further questions the use of “single shot” WB-CT obtained by the time of diagnosis, as baseline MRI or PET/CT has to be obtained before treatment as reference study for later assessment of response and MRD.

## MRI

Morphologic sequences covering the axial skeleton or the whole body, dynamic contrast-enhanced (DCE) examinations and diffusion weighted images (DWI) have been developed to assess bone marrow involvement and its response to therapy [7, 49, 50].

### Examination design

#### Anatomic sequences

**Fig. 2:**
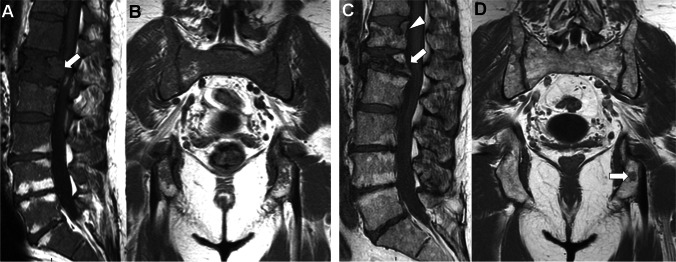
52 year-old patient with newly diagnosed MM (IgA lambda, stage IIIA, ISS 3): initial and post-ASCT axial skeleton MRI findings. A, B Baseline T1- weighted sagittal MR image of the lumbar spine (A) and coronal image of the pelvis (B) performed for the workup of acute lumbar pain show abnormal diffuse low signal intensity of the bone marrow, with pathologic fracture of the L1 vertebral body with mass effect on the spinal canal (arrow in A), leading to the diagnosis of MM. C, D Corresponding MR images obtained after high dose cytotoxic treatment and ASCT show return to normal signal intensity of the bone marrow, decrease in the mass effect of the L1 vertebral compression fracture (arrow in C). Note the appearance of a new vertebral fracture involving the upper endplate of the T12 vertebral body (arrowhead in C), and right ischiatic focal lesion suggesting residual abnormalities (arrow in D)

The first MRI examinations targeting the study of the bone marrow in MM covered the “axial skeleton”, i.e. the spine and pelvis [51, 52]. This “axial skeleton approach” is still recommended when WB-MRI cannot be obtained for technical reasons or time considerations (Figs. 1,2). WB-MRI examinations covering the body from “eyes to thighs” using 2D image obtained in the coronal and sagittal planes were developed thanks to table mobility, improved coils and development of rapid sequences. This whole body approach extends the diagnostic value of MRI compared with more limited skeletal coverage [53–55]. The development of 3D T1-weighted images using either fast spin echo or gradient echo Dixon techniques avoids repetition of 2D sequences in different planes as they allow multiplanar reformat, in particular sagittal views to study spinal complications [56, 57].

**Fig. 3:**
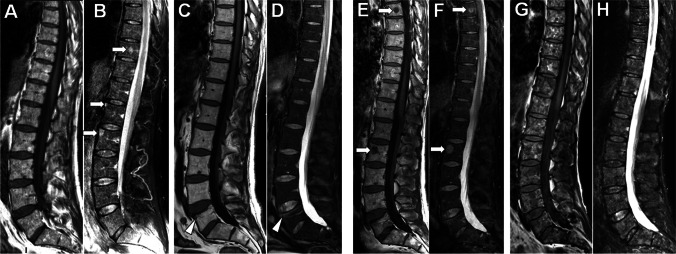
53 year-old man with newly diagnosed MM (IgG A kappa, stage III, ISS 1): MRI findings at baseline, post ASCT, and at relapse. A, B Baseline sagittal T1- (A) and STIR-weighted (B) MR images of the whole spine show diffuse heterogeneous and relatively low signal intensity of the bone marrow on T1, with heterogeneous signal and high signal intensity foci best seen on STIR (arrows in B). C, D Corresponding MR images obtained 12-m later after high dose cytotoxic treatment and ASCT (complete biological response) show complete return to normal signal intensity of the bone marrow. Note the appearance of a new “benign looking” vertebral fracture involving the L5 vertebral body (arrowhead in C and D). E, F Follow-up sagittal T1- (E) and STIR-weighted (F) MR images obtained 6-m later during maintenance therapy show reappearance of several foci of low/high signal intensity (arrows in E, F). G, H Follow-up sagittal T1- (G) and STIR-weighted (H) MR images obtained 12-m later after second line treatment show disease progression with severe increase in marrow infiltration and focal lesions

**Fig. 4:**
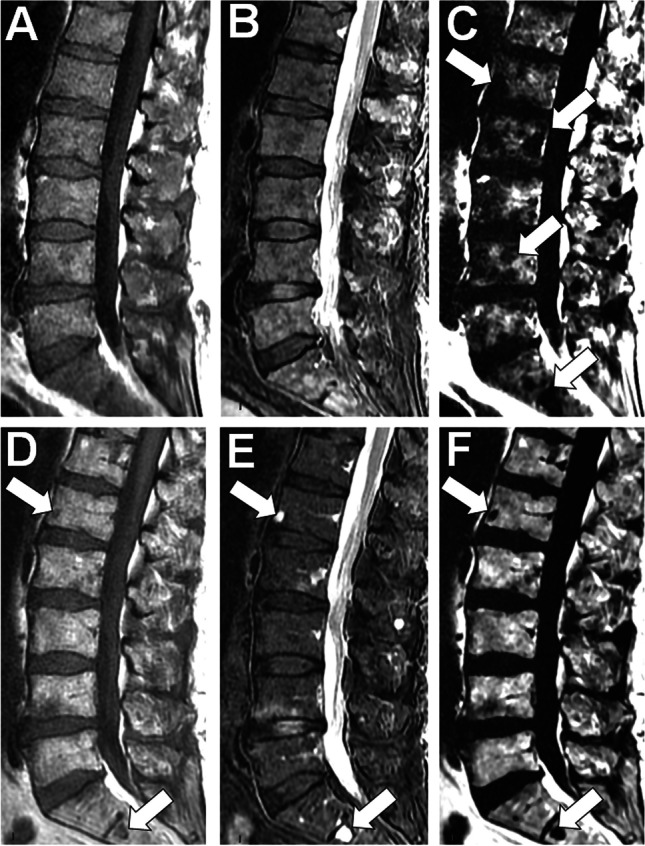
73 year-old man with newly diagnosed MM (IgG G kappa, stage III, ISS 1): MRI findings at baseline and post ASCT. A, B, C Baseline sagittal T1- (A) and T2 Dixon Water Only (B) and Fat Only (C) MR images of the lumbar spine show diffuse heterogeneous and relatively low signal intensity of the bone marrow on T1, with heterogeneous nodular high signal intensity on the Water Only image. Foci of marrow replacement are evident on the Fat only image (arrows in C), underestimated on the T1 image probably to the relatively high signal of myeloma foci on this sequence. D, E, F Post-treatment corresponding MR images obtained 12-m later after high dose cytotoxic treatment and ASCT show almost complete return to normal signal intensity of the bone marrow on the different sequences. Note however the persistence of rare foci of low signal on the T1 and Fat Only images and very high signal on the Water Only image (arrows in D, E, F)

Regarding sequences, axial skeleton and WB-MRI studies first relied on the combination of T1 and STIR/ fat saturated T2 sequences [51]. The latter “fluid sensitive-fat saturated” sequences are mandatory as MM lesions may present a relatively high signal intensity and may be overlooked on T1-weighted images [49, 58] (Fig. 3). These images are now preferably acquired using the Dixon method, as this technique not only provides fat-saturated T2 or STIR equivalent “water only” images but also T2-like images allowing optimal study of the spinal canal and spinal cord and most importantly “fat only” images providing T1-like information and unrivalled detection of focal lesions on a background of fatty marrow, questioning the residual need for T1 images [58–60] (Fig. 4). This T2 Dixon approach has recently been extended at the scale of the whole body [61]. Beside anatomical images, the Dixon technique allows the calculation of the marrow fat fraction (FF) which is gaining interest along with ADC measurements as a biomarker for response evaluation. Indeed, the fat proportion is expected to increase in focal lesions and diffuse marrow infiltration in response to treatment [62].

#### Diffusion-weighted imaging (DWI)

DWI sequences have been introduced in WB-MRI examinations for almost a decade [63–66]. DWI MRI is the most sensitive technique for the detection of bone marrow lesions, with a higher sensitivity than CT and FDG-PET/CT, especially in patients with either subtle or severe diffuse infiltration [67]. It is also the sequence of choice to evaluate treatment response, especially in late stage disease.

Interpretation of DWI relies on the combined evaluation of reconstructed morphological images acquired at different b values (using MPR and MIP approaches, often read in inverted greyscale) and quantitative ADC maps derived from these acquisitions. Due to the presence of large hydrophobic fat cells, limited extracellular space, low water content and lower vascularity, the normal bone marrow has very low ADC values, ranging from 0.2 to 0.5 × 10^–3^ mm^2^/s in the spine and pelvis [68]. Focal MM lesions and diffuse marrow infiltration present increased ADC values compared with those of the normal marrow due to higher cellularity, decrease in fat content and higher vascularity. Mean ADC values of 0.36, 0.77 and 1.046 × 10^–3^ mm^2^/sec were measured in areas of normal appearing marrow, focal lesions and diffuse marrow infiltration, respectively, in MM patients [69]. Focal bone marrow lesions and diffuse severe infiltration by MM appear as focal or diffuse areas of increased signal intensity on low (0–100 s/mm^2^) and most specifically on high (500–1000 s/mm^2^) b value DW images, compared with normal bone marrow and adjacent muscles. These morphologic DWI sequences should be read in parallel with T1 and STIR or T2 Dixon images and with ADC maps to reach optimal diagnostic value and avoid some false positive findings [51, 70].

### Response assessment with MRI

The assessment of response should rely on the acquisition and careful comparison of exhaustive MRI examinations obtained before and after treatment on the same MRI magnet using the same protocol [71]. Detailed clinical information must be available, including time of initial diagnosis, serum paraprotein and light chain levels, last trephine status, minimal residual disease status, symptomatic sites and suspicion of cord or nerve root compression, current treatment, history of marrow transplant and details of additional treatments (radiotherapy, surgery, marrow stimulating factors, …).

#### Assessment of response on morphologic sequences

**Fig. 5:**
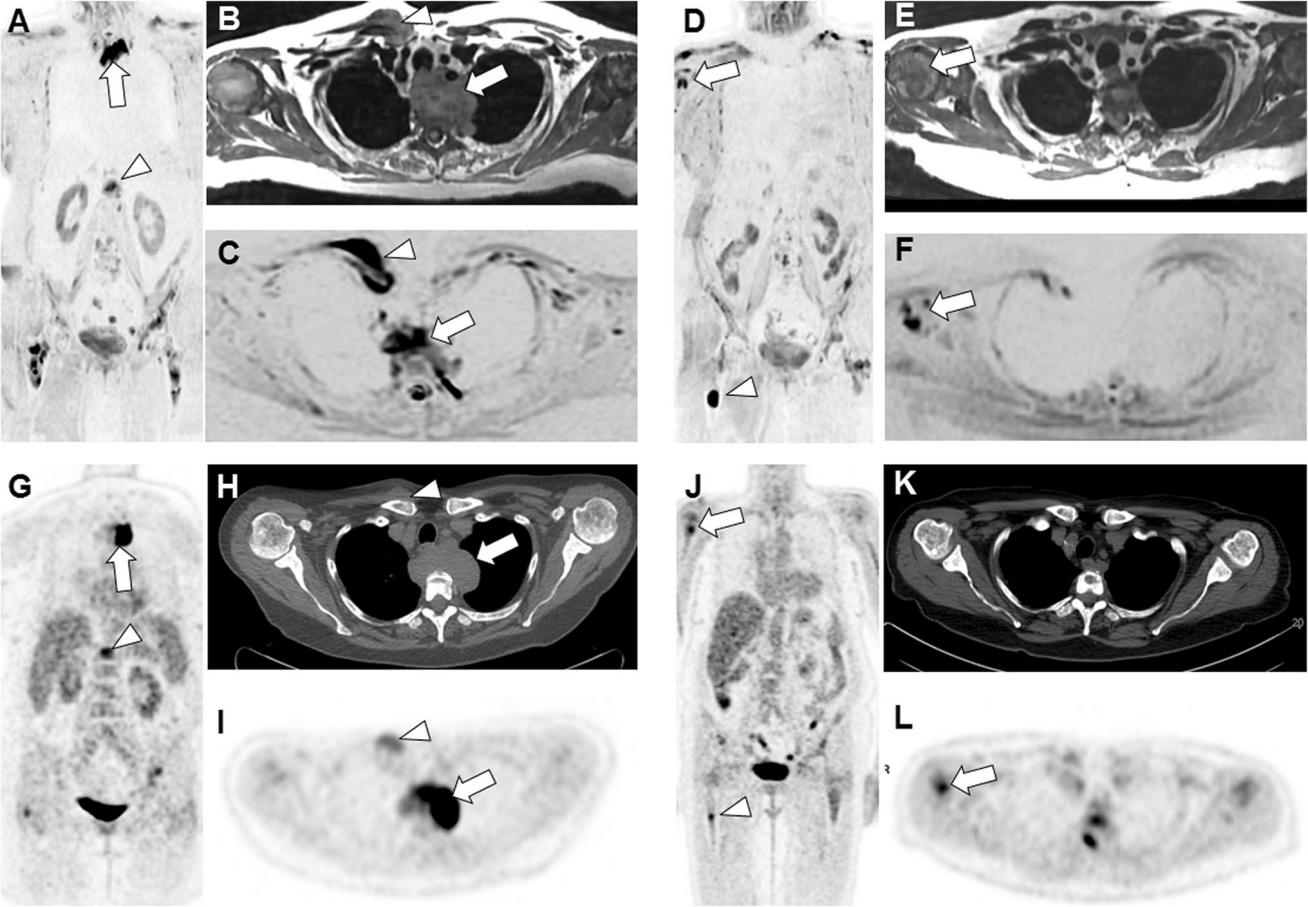
74 year-old woman with MM (IgG lambda stage IIIA, ISS 1 MM with adverse cytogenetics (del 17p), fourth line (salvage) therapy with Elotuzumab, Lenalidomide, and Dexamethasone): WB-MRI and PET/CT performed for evaluation of disease and treatment response after 3 cycles, biological progression. WB-MRI and PET show bone and extramedullary disease, heterogeneity of response and residual disease. A-C Baseline MRI findings. Coronal DWI MR image (B = 1000 s/mm^2^) (A) shows active bone lesions within both femurs, within the T12 vertebra (arrowhead), and paramedullary mass in the superior and anterior mediastinum (arrow). Transverse T1-weighted (B) and DWI (C) MR images confirm the presence of para-medullary extension adjacent to upper thoracic vertebra (arrow) and to the medial portion of the right clavicle (arrowhead). D-F: Post-treatment MRI findings. Coronal DWI (D), and transverse T1 and DWI MR images show major response of almost all lesions, including the paramedullary extension in the mediastinum and right clavicular regions. The coronal image and the transverse images show new lesions within the right humerus (arrows in D-F) and right femur (arrowhead in D). G-I Corresponding PET/CT findings. Coronal PET image shows exactly the same findings: femoral, T12 (arrowhead) and mediastinal lesions (arrow)(SUVmax of femoral, T12 and mediastinal lesions:: 7.3, 16.9, 12.7; mediastinal blood pool: SUVmax 2.1). Corresponding transverse CT (H) and PET (I) images show soft tissue lesion adjacent to an upper thoracic vertebra (arrow) and to the medial part of the right clavicle (arrowhead). J–L Follow-up PET/CT findings. Corresponding PET/CT images show exactly the same findings: major response of almost all lesions including paramedullary extension in the mediastinum and right clavicular region, but appearance of new foci within right humerus (arrows in J and L) and femur (arrowhead in J) (SUVmax of femoral, T12 and mediastinal lesions: 4.3, 4.6, 4.3; new humeral lesion: SUVmax 5.6; new femoral lesion: SUVmax 5.3; mediastinal blood pool: SUVmax 2.3)

Bone marrow infiltration by MM may present a focal, a diffuse and a “salt and pepper” patterns, although the bone marrow may also keep a normal appearance [50, 72]. These patterns and lesion number and size should be carefully evaluated on anatomic fat and fluid sensitive sequences and on morphologic high b value DWI images, and compared between pre- and post-treatment examinations. Decrease in focal lesion number and size and return from diffuse or focal patterns of marrow infiltration to a normal marrow appearance indicate response and are of prognostic significance [73] (Figs. 1–4). Additional qualitative signs of response are useful, particularly the progressive appearance of a “fatty halo” at the periphery of regressing focal lesions and the decrease in size of extraosseous extension of some lesions (paramedullary lesions) [74] (Fig. 5). Conversely, evolution from a normal marrow appearance to focal or diffuse patterns, increase in number and size of focal lesions, occurrence of malignant vertebral compression fractures, appearance/extension of extraosseous spread or soft tissue plasmocytomas indicates progressive disease [74] (Figs.3, 5).

Responsive MM lesions usually show a progressive decrease in signal intensity on fat saturated T2/STIR images on follow-up MRI examinations [45]. However, some responsive focal lesions may remain visible as high signal foci, sometimes for years presumably due to cystic/necrotic transformation [75, 76] (Fig. 4). The same phenomenon can be observed on DWI, where those necrotic lesions can stand out on high b value images due to the “T2 shine through” phenomenon but are recognized on ADC maps thanks to their very high ADC values.

#### Fat fraction (FF)

Early change in FF after induction of chemotherapy has been demonstrated as a significant predictor of the depth of response: two studies showed a significantly higher increase in the marrow FF in responders (complete response and very good partial response) compared with non-responders (partial response, stable disease, progressive disease) early after initiation of treatment [77, 78].

#### Assessment of response on DWI sequences

**Fig. 6:**
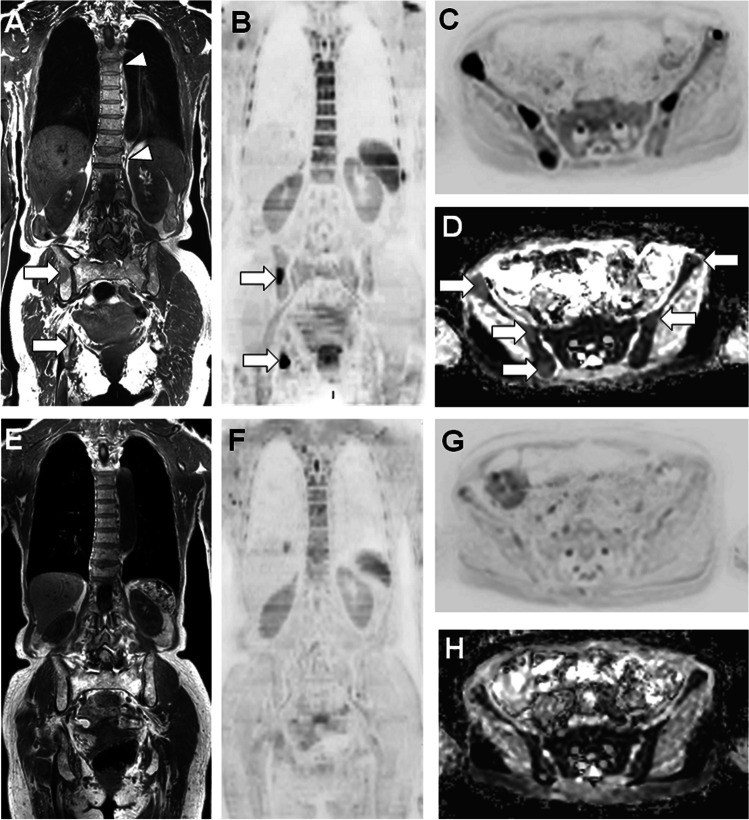
80-year-old woman with MM (IgG lambda stage IIIA, ISS 1, adverse cytogenetics (t(14;16)) – VGPR after 8 cycles of VCD): WB-MRI performed for evaluation of treatment response shows complete imaging response with no residual disease. A-B Baseline MRI findings. Coronal T1 (A) and DWI (B = 1000 s/mm^2^) (B) MR images show active bone lesions within the right pelvic region (arrows), and benign appearing vertebral fractures (arrowheads). C–D Transverse DWI (B = 1000 s/mm^2^) image (C) and ADC map (D) within the pelvis show presence of 3 lesions on the right and 2 on the left. ADC in the lesions was 750 mm^2^/s, Vs 480 mm^2^/s in the adjacent marrow. E–F Post-treatment MRI findings. Coronal T1 (E) and DWI (B = 1000 s/mm^2^) (F) MR images show disappearance of all bone lesions. G–H Transverse DWI image (G) and ADC map (H) show disappearance of pelvic lesions and return to normal of ADC values

Aside from morphologic signs of disease response or progression observed on high b value images, quantitative ADC measurements inform on disease activity and on global tumor burden [66, 79] (Fig. 6). DWI is of particular value in the presence of stable disease on anatomical sequences, which may present delay in observing disappearance of marrow lesions, and in advanced disease, where scar or necrotic inactive tissue may show persistent signal abnormalities on fat and fluid sensitive sequences, being difficult to differentiate from residual active lesions.

**Fig. 7:**
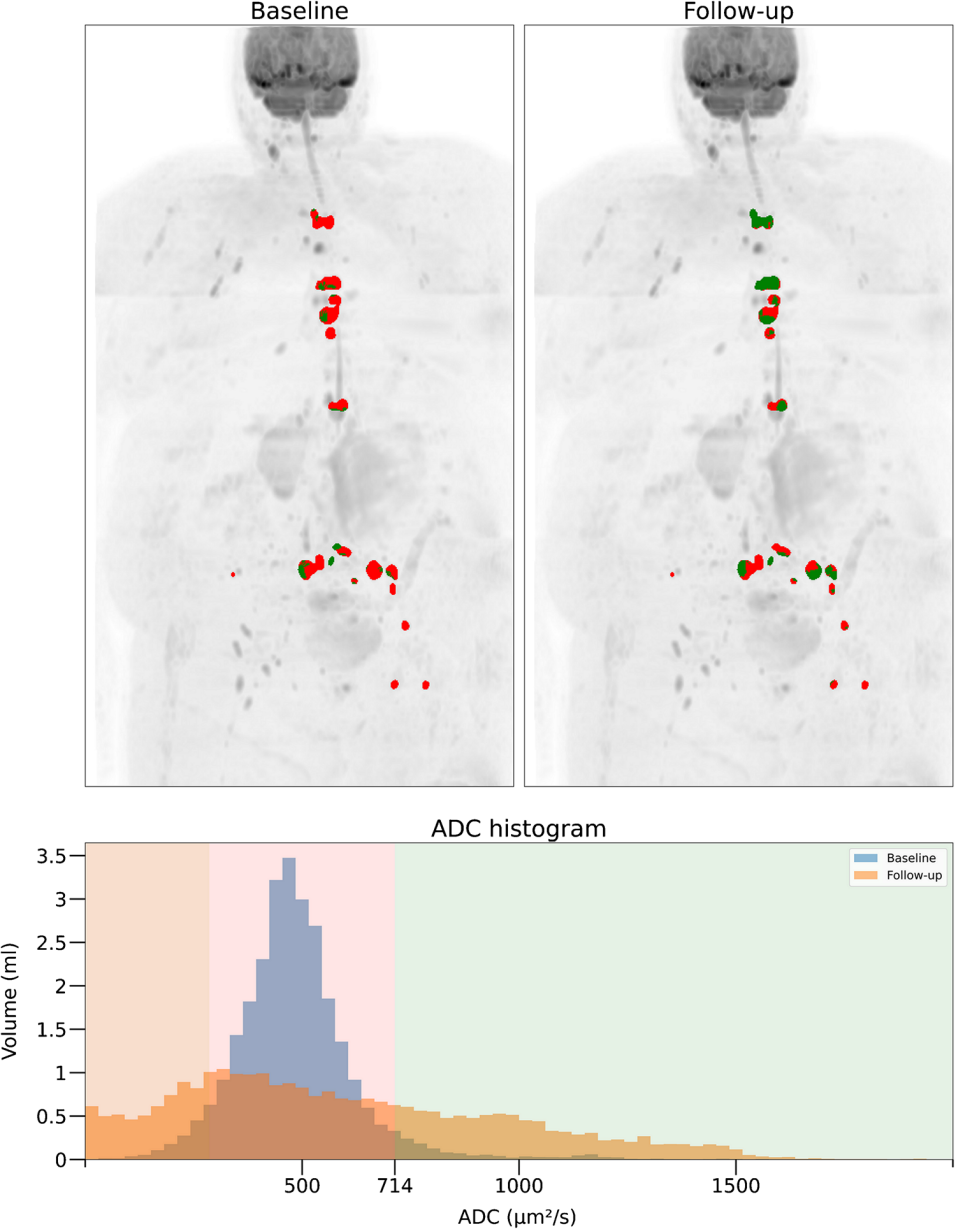
Quantitative evaluation of response by DWI lesion volume measurement in the spine in a 78 year-old woman with advanced MM. The biological response as assessed using the IMWG criteria was qualified as “partial response”. Baseline (Top left image) and follow-up (Top right image) data. After semi-automated segmentation (exclusion of extraskeletal signal using signal intensity threshold and skeletal mask), focal MM lesions are projected on MIP views of the B1000 DWI images. If a voxel within the lesions has an ADC value ≥ 1100 µm2/s, the projection image gets a green colour. Red coloured voxels have lower ADC values and represent untreated disease. Green coloured voxels replacing red ones on the follow-up image represent voxels that are ‘likely’ to be responding. The follow-up ADC image indicates an increase in ADC and thus a response to treatment, although some red voxels remain present, indicating residual active disease. The « spinal» volume of abnormal ADC was estimated to 24.2 mL of bone marrow before therapy and 3.75 mL after therapy. Comparison of pre- and post-treatment histograms (Bottom image) shows both lower and higher ADC values after treatment. This reflects return to normal bone marrow (lower ADC) and tumour cell kill (higher ADC), respectively.

Changes in ADC are measured using ROI placed within a lesion or within diffusely infiltrated marrow on ADC maps of two consecutive MR examinations. These measurements in MM lesions have shown good reproducibility with a coefficient of variation reported to be less than 3% [66]. After treatment, an initial increase in mean ADC values is observed in responding lesions but also in the whole skeleton in responding patients and is absent in nonresponders [66]. This phase is followed by a delayed decrease in ADC values, present at 20 weeks after chemotherapy, reflecting return to normal marrow composition and fat content [65] (Fig. 6). Analysis of ADC histograms further reinforces this evaluation of response to treatment, showing progressive displacement to lower values and flattening in responsive disease, indicating decreased cellularity and return of marrow fat, while ADC histograms before and after treatment in nonresponders show little change in both values and shape [66](Fig. 7).

Beside ADC evaluation in individual lesions or in diffusely infiltrated marrow, a total diffusion volume (TDV) and global ADC (gADC) can be calculated to figure the total lesion burden, both at baseline and on follow-up WB-MRI studies. After manual or (semi-)automated segmentation, the tumor volume is determined by measuring the quantity of voxels presenting a range of ADC values defined as abnormal [80]. Although being still work-in progress, these quantitative approaches have shown excellent inter- and intra-observer reproducibility [81] (Fig. 7).

#### Multiparametric approach and guidelines for response assessment

To assess response, anatomic sequences, FF images and DWI sequences should be studied altogether using the image coregistration and scrolling capabilities of PACS workstations. Pre- and post-treatment findings should be compared using morphologic and quantitative approaches. Increase in marrow FF and ADC values are most discriminant features, preceding and outperforming decrease in the volume of focal lesions to distinguish responders from non-responders patients as determined using the “classical” IMWG response criteria [10, 82, 83].

Standardization of imaging acquisition, reading and response assessment is crucial for generating reproducible results in daily practice and for strengthening the value of WB-MRI for response assessment and prognostication in clinical trials. The Myeloma Response Assessment and Diagnosis System (MY-RADS) provides guidelines regarding the choice, acquisition and interpretation of WB-MRI [71].

It proposes a structured reporting for each individual WB-MRI examination, which should describe the indication (MM, ND or follow-up), the acquisition protocol, the marrow infiltration pattern (normal, focal, focal and diffuse, diffuse, variegated), the location and size of five “dominant” focal bone lesions and of paramedullary or extramedullary sites. Most importantly, this system proposes 5 “response assessment categories” (RAC) that should be used for homogeneous and reproducible response evaluation in bone lesions [71]. Those RAC use a 5-point scale defined hereafter (Table 1). For “soft-tissue disease”, either extramedullary (which is observed in approximately 3–10% of patients at diagnosis but presents an increasing frequency raising to more than 20% of patients in advanced stages) or paramedullary (20–30% of patients), size criteria are used following Response Evaluation Criteria in Solid Tumors (RECIST) 1.1 [11, 84] (Fig. 5).

**Table 1:** The MY-RADS response assessment categories (RAC) based on comparison between pre- and post-treatment WB-MRI/DWI studies performed in MM patients (after Messiou et al. [71]).

Response categories	Morphological changes	Multiparametric data
Highly likely response (RAC 1)	-Diffuse/focal → Normal pattern -Focal lesions: ↓ number/size	-↓ SI of focal lesions or diffuse infiltration on high b-value images (relative to muscles) -ADC ↑ > 40% -ADC ↑ from < to > 1400 μm2/s
Likely response (RAC 2)	-Focal lesions: limited ↓ number/size	-↑ ADC from baseline > 25% but < 40%, and ADC increase from < 1000 to < 1400 μm2/s
Stable disease (RAC 3)		
Likely progression (RAC 4)	-Focal lesions: equivocal ↑ number/size -Reappearance/enlargement of previously responsive lesions -Spinal canal infiltration without neurologic compression	Absence of size change but ↑ in SI of focal lesions on high b-value images (with ADC < 1400 μm2/s)
Highly likely progression (RAC 5)	-Normal /focal → Diffuse pattern -↑ focal lesion number/ size -Appearance of pathological fractures or neurologic compression -New or increased soft tissue extension	-New lesions of high signal intensity on high b-value images with ADC value > 600 and < 1000 μm2/sec

RAC (response assessment category) should be evaluated in each anatomic region (cervical, thoracic, lumbar spine, pelvis, long bones, skull, ribs) and summarized for the whole skeleton. Abbreviations: *ADC* average diffusion coefficient, *SI* signal intensity.

### Role of MRI to assess minimal residual disease (MRD)

As a preliminary remark, the term “MRD”, often used in the literature to define residual abnormalities seen on PET or MRI studies performed in treated MM patients, is probably abusive and does not situate at the same scale as the term MRD when used for defining molecular signs of disease using NGF or NGS. The term “imaging residual disease” would probably be more appropriate.

Combined drug treatment strategies and ASCT have strongly improved response with > 50% of MM patients achieving CR by conventional criteria. Serum markers and bone marrow examinations are insufficient to identify MRD, which frequently persists in CR patients [9]. Advanced molecular techniques allow sensitive detection of MRD but rely on bone marrow aspirates and are limited by the heterogeneous distribution of clonal plasma cells within the skeleton and by the eventual occurrence of extramedullary lesions.

WB-DWI and FDG-PET/CT may overcome some of the limitations of molecular techniques and have been used to redefine CR in patients with MRD-negative bone marrow.

Several studies have demonstrated that the observation of residual lesions on MRI studies performed after completion of high-dose chemotherapy protocols was associated with an adverse outcome [73, 85]. Recent works demonstrated the ability of WB-MRI/DWI sequences to detect MRD in MM [86]. Comparative studies even suggest that WB-MRI/DWI might be equivalent or even slightly superior to PET/CT in the assessment of MRD in MM [11, 87]. This comparison will be addressed below.

### Particularities and pitfalls in response evaluation

#### Heterogeneity of response

Careful attention should be paid to all individual lesions as heterogeneity in behaviour of these lesions under treatment is a hallmark of cancer and of MM in particular [88]. WB-MRI, by providing an extensive skeletal and extraskeletal coverage and exhaustive follow-up of lesions, can capture this discordant feature which may be an early indicator of treatment failure and progressive disease [71] (Fig. 5).

#### Vertebral compression fractures (VCF) and neurologic compression

The observation of a new VCF during MRI follow-up of MM patients is frequent and should only be considered as a sign of progressive disease if this fracture presents malignant characteristics, as defined in the literature, i.e. marrow replacement, mass effect, posterior vertebral elements or soft tissue extension [89, 90]. Indeed, “simple” osteoporotic (benign appearing) VCF are extremely frequent in MM, in relation to the diffuse osteopenia induced by the disease and its treatments, especially corticoids. An MRI study showed that only 33% of VCF observed in MM appear malignant, while 67% of VCF are benign appearing [12, 91]. This distinction is very important during follow-up of patients in biochemical remission with no other signs of progression [92] (Fig. 2). MRI identifies benign fractures that should not be considered as “skeletal related events” indicating disease progression and requiring treatment change. In tumoral VCF, MRI is the reference method for assessing spinal cord and/or nerve compression and for evaluating the need for surgical decompression, stabilization or local radiation therapy.

### Dynamic contrast-enhanced (DCE) MRI

**Fig. 8:**
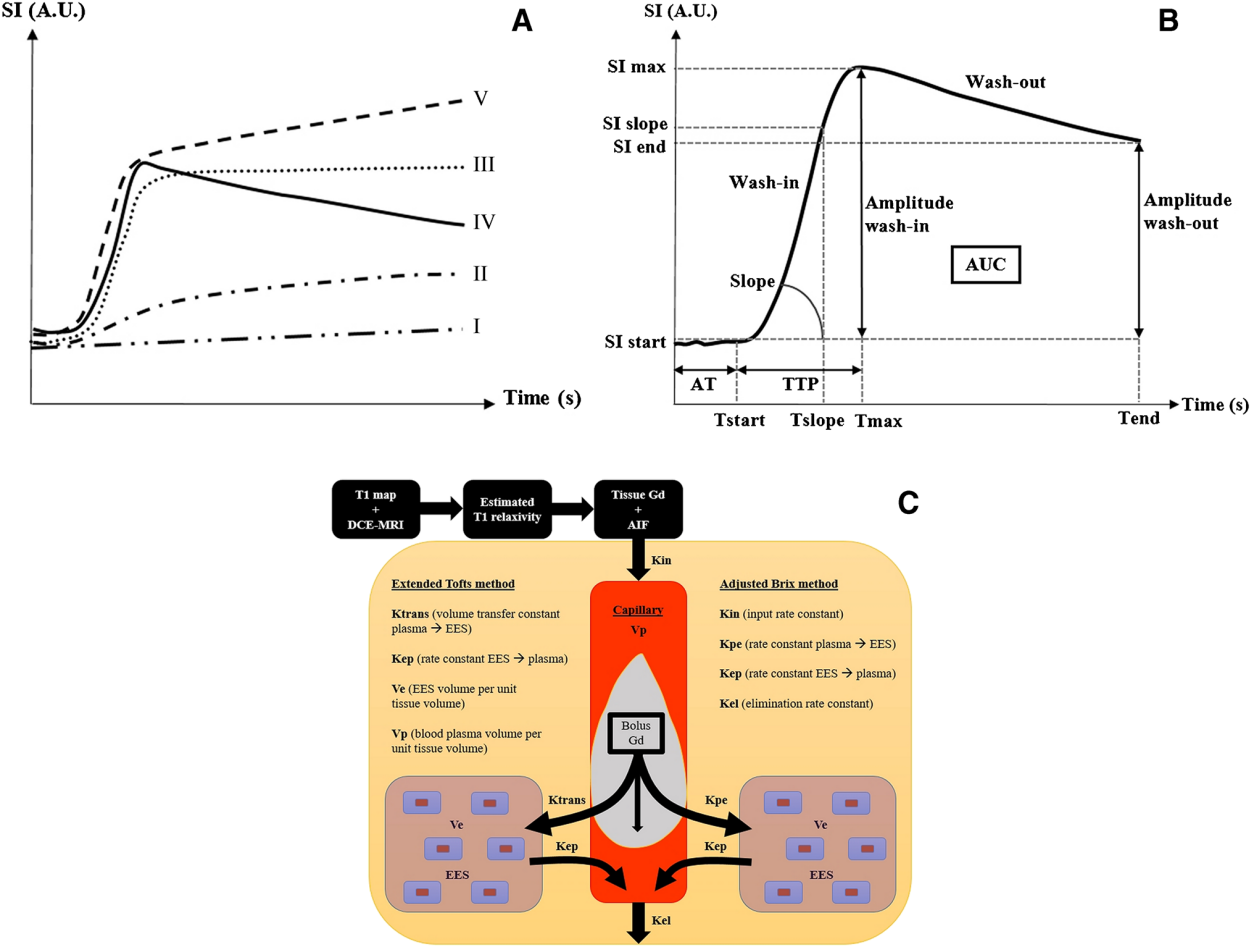
Overview of DCE-MRI interpretation. A Curve types for visual assessment of disease activity. Curve upscale on follow-up MRI is indicative of progression, therapy failure or relapse. Curve downscale is indicative of response to therapy. Types III/IV/V have a steep wash-in and first pass (‘active curve types’: high perfusion, high tissue vascularization, low capillary resistance, high capillary permeability). Type III: plateau. Type IV: rapid wash-out (small interstitial space, high cellularity). Type V: continuous wash-in (large interstitial space). Types I/II have a low wash-in and first pass (‘inactive curve types’: low perfusion, low tissue vascularization, high capillary resistance, low capillary permeability). Type I: very low or no enhancement. Type II: slow sustained enhancement. SI = signal intensity. A.U. = arbitrary units. B Semi-quantitative analysis. Descriptive parameters of the time intensity curve (TIC). SI = signal intensity. A.U. = arbitrary units. T = time. TTP = time to peak. AT = arrival time. AUC = area under curve. s = second. C Quantitative analysis flowchart. The tissue gadolinium concentration is calculated from the estimated T1 relaxivity changes based on information of the combined measured T1 map and the dynamic scan. Calculation of quantitative gadolinium distribution parameters can be done by fitting the tissue gadolinium concentration and the arterial input function data in the pharmacokinetic extended/adjusted models of Tofts or Brix. Ktrans, Kep, Kin, Kpe and Kel are equilibrium constants describing the distribution of gadolinium contrast medium over the vascular, interstitial and cellular compartments. Ve is the EES volume per unit of tissue volume. Vp is the blood plasma volume per unit of tissue volume. DCE-MRI = dynamic contrast-enhanced MRI. Gd = gadolinium contrast medium. AIF = arterial input function. EES = extracellular extravascular space. Blue rectangles = cells in tissue e.g. plasma cells in bone marrow.

Dynamic contrast-enhanced (DCE) MRI evaluates tissue vascularization, perfusion, capillary resistance and permeability in a limited portion of the skeleton by time series of images obtained after intravenous injection of gadolinium [93]. A multi-slice fat saturated ultrafast T1-weighted sequence is performed after injection. Postprocessing consists in manually placing regions of interest (ROI) in the diseased and reference tissue (e.g. muscle or artery) to acquire time-intensity curves (TIC) in which the signal intensity is plotted against time points. Evaluation of these curves is done either qualitatively (Fig. 8a) by visually determining the curve pattern within the ROI (I-V) or semi-quantitatively by calculating descriptive parameters of the TIC (signal intensities with corresponding time points, absolute and relative enhancement of a vertebra compared to muscle, slope wash-in, slope wash-out, area under curve) (Fig. 8b) [94]. By applying pharmacokinetic models, a time-concentration curve can be derived. Tissue and contrast distribution parameters over the vascular/cellular/interstitial compartments can be derived with corresponding parametric maps (Fig. 8C) [94]. Parametric histograms and changes of the quantitative parameters before and after therapy can be analysed [95].

**Fig. 9:**
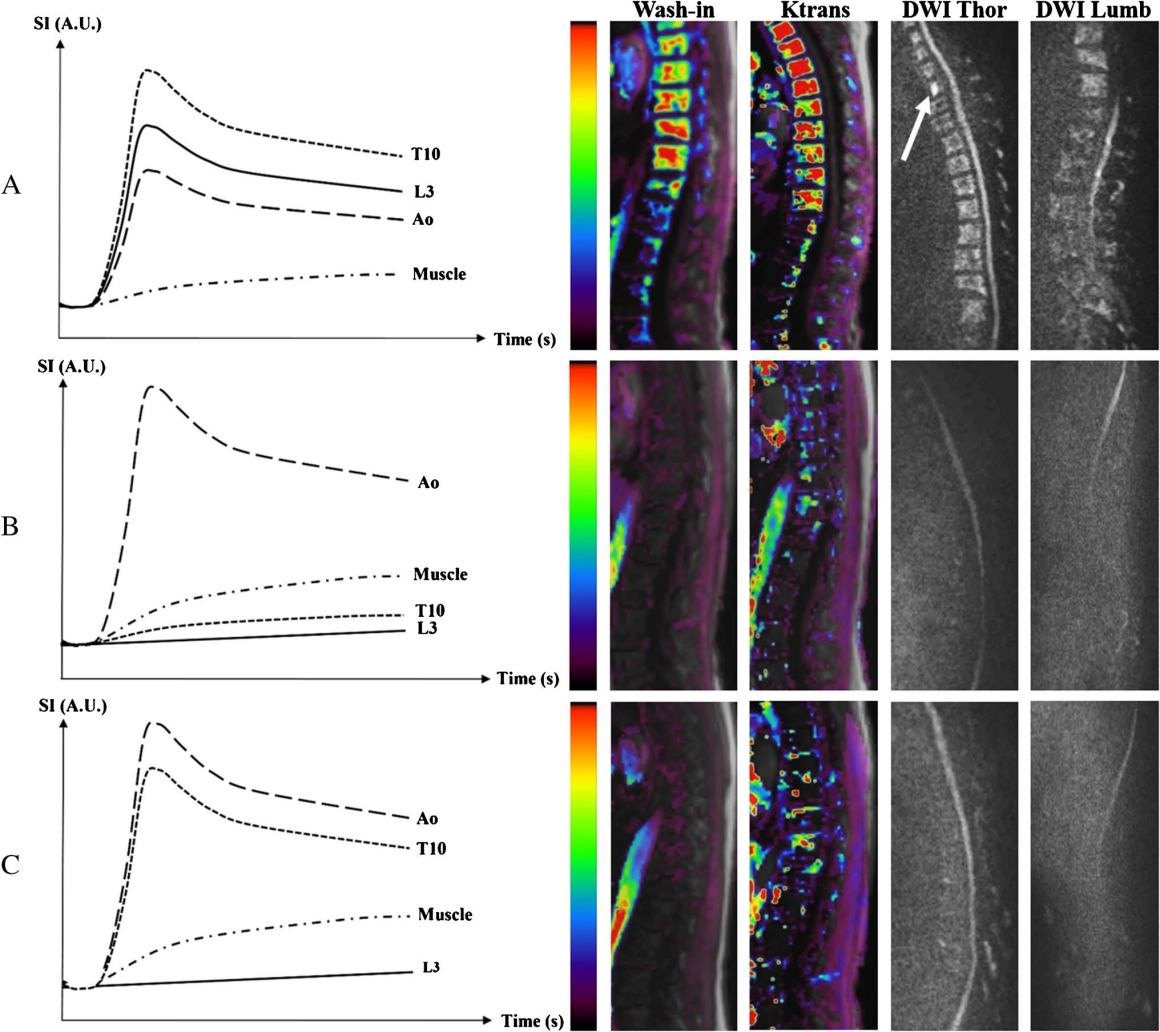
Combined DWI and DCE-MRI in a 59 year-old female with ND MM (IgA lambda, ISS-1, 60% bone marrow plasmocytosis). A parasagittal DCE-MRI of the thoracic and lumbar spine is classically performed with ROIs drawn in the aorta, a paravertebral muscle, T10 and L3 vertebrae to obtain the TICs. In the aorta, the ROI lies near the middle of the vessel in a zone without flow void artefacts. In vertebrae, the ROI lies near the middle of the vertebral body with exclusion of the entrance of vertebral vessels, endplates and other pathologies. Left to right: Curve types, parametric map scale, wash-in slope and Ktrans parametric maps, thoracic and lumbar DWI. A DWI and DCE-MRI at ND MM: DWI (b1000)—thoracic and lumbar images show high diffusion restriction in all vertebrae with a focal lesion (white arrow) in the T1 vertebra. DCE-MRI—thoracic and lumbar vertebrae show active type IV curves with steep wash-in, high amplitude and high wash-out rate. Slope wash-in: 0,95. Ktrans: 0,83. B DWI and DCE-MRI at complete response 6 months post-ASCT with high dose melfalan after 4 cycles of VTD induction therapy (bortezomib, thalidomide, dexamethasone). DWI (b1000): normal diffusion pattern in the whole spine. DCE-MRI: thoracic and lumbar vertebrae show inactive type I curves. Slope wash-in: 0,027. Ktrans: 0,15. C DWI and DCE-MRI by the time of relapse/progressive disease 2 years after successful ASCT and 1 year of lenalidomide maintenance therapy. DWI (b1000): all vertebrae show intermediate diffusion restriction without focal lesions. DCE-MRI: thoracic active type IV curve and lumbar inactive type I curve (attributed to fatty infiltration post-ASCT and maintenance therapy). Slope wash-in: 0,056. Ktrans: 0.21

Active curves obtained within lesions in ND MM patients are characterized by type IV and V but mostly type IV [94]. Using the latest IMWG response criteria, Dutoit et al. found a “downgrade” in active curve types from 96 to 32% in responders (Fig. 9). The paradoxically active curve types in patients with CR (mostly type III) can be explained by increased vascularization in hyperplastic reactive red bone marrow, either physiologically in younger adults or reactively after marrow stimulating therapy [94]. In CR, a decrease of 73% in wash-in slope and 71% in absolute enhancement can be observed, indicative of tumoral vessel destruction by therapy [96].

Lin et al. highlighted the value of the parameter maximal percentage of bone marrow enhancement, where a post-induction chemotherapy value of more than 96.8% identified poor responders with a sensitivity of 100% (76.9% specificity, area under ROC curve 0.90). The maximal percentage of focal lesion enhancement did not significantly differ between good and poor responders, but the time point of maximum enhancement was delayed in good responders [97]. Quantitatively, responders had significantly lower amplitude A and Kep values after therapy compared with non-responders, whereas the latter showed significantly higher values of amplitude A before the start of therapy as compared with responders. Higher values of amplitude A before the start of therapy are a poor prognostic factor for therapy success [97]. As the response assessment in diffusely infiltrated bone marrow is far more complex than in focal lesions, larger regions of interest analysis (e.g. vertebral body versus reference tissue) using DCE-MRI may be useful to assess treatment response in the absence of focal lesions [94].

DCE-MRI can be combined with anatomical and DWI in a “combined skeletal score”, which summarizes MRI information in one quantitative score to assess therapy response and has shown good correlation with the IMWG response criteria [96]. Interestingly, disease progression and relapse in patients initially classified as clinically good responders can be detected (angiogenic switch anticipating clinical endpoints), suggesting a potential interest of DCE-MRI to detect residual disease after therapy [97].

Despite these promising results, DCE suffers lack of clinical validation, poor reproducibility due to poor standardization of imaging protocols and difficult interpretation. Hence, DCE-MRI is not included in the current imaging guidelines in MM [37, 71].

## PET/CT

### Principles

Positron emission tomography with computed tomography using [^18^F]fluorodeoxyglucose (FDG-PET/CT) is a whole-body imaging modality widely used for tumour detection/staging, monitoring treatment response and predicting prognosis in a variety of malignancies. FDG-PET/CT uses a radiolabelled glucose analogue in which the C-2 hydroxylgroup is replaced by the positron emitting fluorine-18 atom [^18^F]. After intravenous administration, [^18^F]FDG and glucose are transported across the cell membrane through facilitative glucose transporters and once intracellularly, they are phosphorylated by hexokinase to [^18^F]FDG-6-phosphate and glucose-6-phosphate, respectively. In contrast to the latter, [^18^F]FDG-6-phosphate is not further metabolized in the glycolytic pathway because of the [^18^F] substitution and the molecule consequently gets trapped intracellulary allowing an accurate assessment of glucose metabolic activity [98]. The CT component of the examination allows the anatomic localization of metabolic foci and the detection of lytic lesions, whether or not these are associated with increased tracer uptake, outperforming conventional radiographs.

### Response measurement and minimal residual disease

FDG-PET/CT is currently the imaging modality of choice for assessment of therapeutic response [37, 99]. The wide implementation of this hybrid modality in routine practice relies on its capability to distinguish active from inactive residual disease and to provide an accurate anatomic mapping of bone and extra-medullary lesions (Figs. 5, 6).

Pioneering work by the University of Arkansas for Medical Sciences has demonstrated that complete [^18^F]FDG suppression in all focal bone lesions before ASCT conferred superior overall survival (OS) and event-free survival (EFS) and that improved survival could be obtained by altering therapy in patients not achieving complete FDG suppression after induction therapy [100]. The powerful prognostic value of FDG-PET/CT after first-line treatment has been confirmed in several prospective studies of ND transplant eligible patients. In 2018, Davies et al. demonstrated in a series of 596 patients that serial FDG-PET/CT assessment post therapy (day 7, post induction, post ASCT and before maintenance therapy) contributed to risk assessment and outcome prediction [101]. As soon as 7 days post-treatment initiation, patients without residual FDG uptake in focal lesions compared with marrow background activity had the same prognosis as those without focal lesions at diagnosis. Moreover, continued suppression of [^18^F]FDG uptake at later time points showed a similar outcome compared with patients without focal bone lesions underscoring that full suppression of focal lesions should be the therapeutic goal [101]. Similar results were obtained by Zamagni et al. in a cohort of 192 ND MM patients after induction therapy and double ASCT. Persistence of residual [^18^F]FDG avid bone lesions after induction therapy defined as a standardized uptake value > 4.2 (a parameter quantifying the degree of glucose consumption) was an early predictor of shorter PFS [102]. Importantly, these findings have been confirmed in a larger patient cohort [103]. Another prospective trial, IMAJEM, compared the prognostic value of [^18^F]FDG-PET/CT and MRI in 134 patients who were randomized to receive a combination treatment with or without ASCT followed by maintenance therapy [104]. [^18^F]FDG-PET/CT normalization (defined as uptake ≤ liver background) before maintenance was associated with better PFS and OS. In contrast, MRI findings (limited to axial skeleton examinations without DWI sequences) before maintenance were not predictive of PFS and OS due to a significant number of false positive results in one study only [104].

The complementarity between functional imaging modalities (FDG-PET/CT or WB-MRI) and bone marrow assessment for detecting MRD has recently been demonstrated by Rasche et al. Among patients with first-line molecular MRD-negative complete remission, functional imaging detected a focal lesion in up to 12% of patients who had a worse PFS [28]. The complementarity of FDG-PET/CT with bone marrow MRD techniques has also been demonstrated in the multicenter phase II randomized FORTE trial in ND transplant eligible MM patients [105]. However, the impact of [^18^F]FDG-PET/CT compared with bone marrow MRD analysis on patient’s outcome warrants further analysis. Finally, the CASSIOPET study showed that PFS was prolonged in patients with PET negativity pre-maintenance (defined as uptake < mediastinal blood pool) who were treated with D-VTd. In addition, more patients treated with D-VTd reached double negativity of PET/CT and MRD post-consolidation in comparison to the group treated with VTd (66.7 vs 47.5%, respectively)[106]. In this study, post-consolidation concordance between MRD and PET-CT was observed in 109/176 (62%) patients with 102 patients being double negative and 7 double positive. These results demonstrate the complementarity between PET and MRD bone marrow techniques and suggest that concordant negativity should be evaluated as a surrogate for outcome prediction.

It is important to emphasize that the definition of complete metabolic response or PET negativity significantly varied across the prospective studies described above, making a head-to-head comparison difficult. The lack of established criteria to define complete metabolic response on FDG-PET/CT has raised the need to standardize and harmonize imaging criteria and to define cut-off values to distinguish negative from positive results. Zamagni et al. performed a joint analysis of 228 ND transplant eligible MM patients enrolled in two independent European prospective randomized phase III trials [107]. All patients underwent FDG-PET/CT at baseline and pre-maintenance therapy and the 5-point Deauville score was applied to describe diffuse marrow and focal lesion uptake. The Deauville score was initially developed for interim PET in lymphoma [108]. The scoring system is detailed in Table 2.

**Table 2:** Summary of the IMPeTUs criteria designed for reading of PET/CT examinations in MM (after Nanni et al.[109])

Lesion type	Site	Number	Grading
Diffuse infiltration	“A” if hypermetabolism in ribs and limbs Skull Spine Extraspinal		5-PS**
Focal lesions		X1 = 0 X2 = 1–3 X3 = 4–10 X3 = > 10	5-PS**
Osteolytic foci		X1 = 0 X2 = 1–3 X3 = 4–10 X3 = > 10	
Fracture on CT	At least one		
Para-medullary lesions	At least one		
Extra-medullary lesions	At least one	Nodal/extranodal *	5-PS**

Notes:

* Anatomic area should be recorded for nodal disease (Cervical, Supraclavicular, Mediastinal, Axillary, Retroperitoneal, Mesenteric, Inguinal) and extranodal disease (Liver, Muscle, Spleen, Skin, Other).

**5-PS = 5-point Deauville scale is used to grade uptake (in diffuse bone marrow infiltration, hottest focal bone marrow lesion, and hottest extramedullary lesion) as follows: 1, No uptake at all; 2, uptake ≤ mediastinal blood pool uptake (SUV max); 3, uptake > mediastinal blood pool uptake but ≤ liver uptake; 4, uptake moderately > liver uptake; 5, uptake markedly > liver uptake (twice or more) and/or any new lesion.

Uni- and multivariate analysis showed that focal lesion and marrow uptake score < 4 at pre-maintenance were associated with prolonged PFS and OS (OS, HR 0.6 and 0.47; PFS HR, 0.36 and 0.24, respectively). These results are in line with the study by Nanni et al. who demonstrated the applicability and reproducibility of the Deauville score, especially score 4 in a cohort of 86 patients (IMPeTUs). Based on these results, liver background has been proposed as reference to identify CR on FDG-PET/CT, but this needs validation in independent prospective trials before refining the definition of PET response criteria proposed by the IMWG [9].

**Fig. 10:**
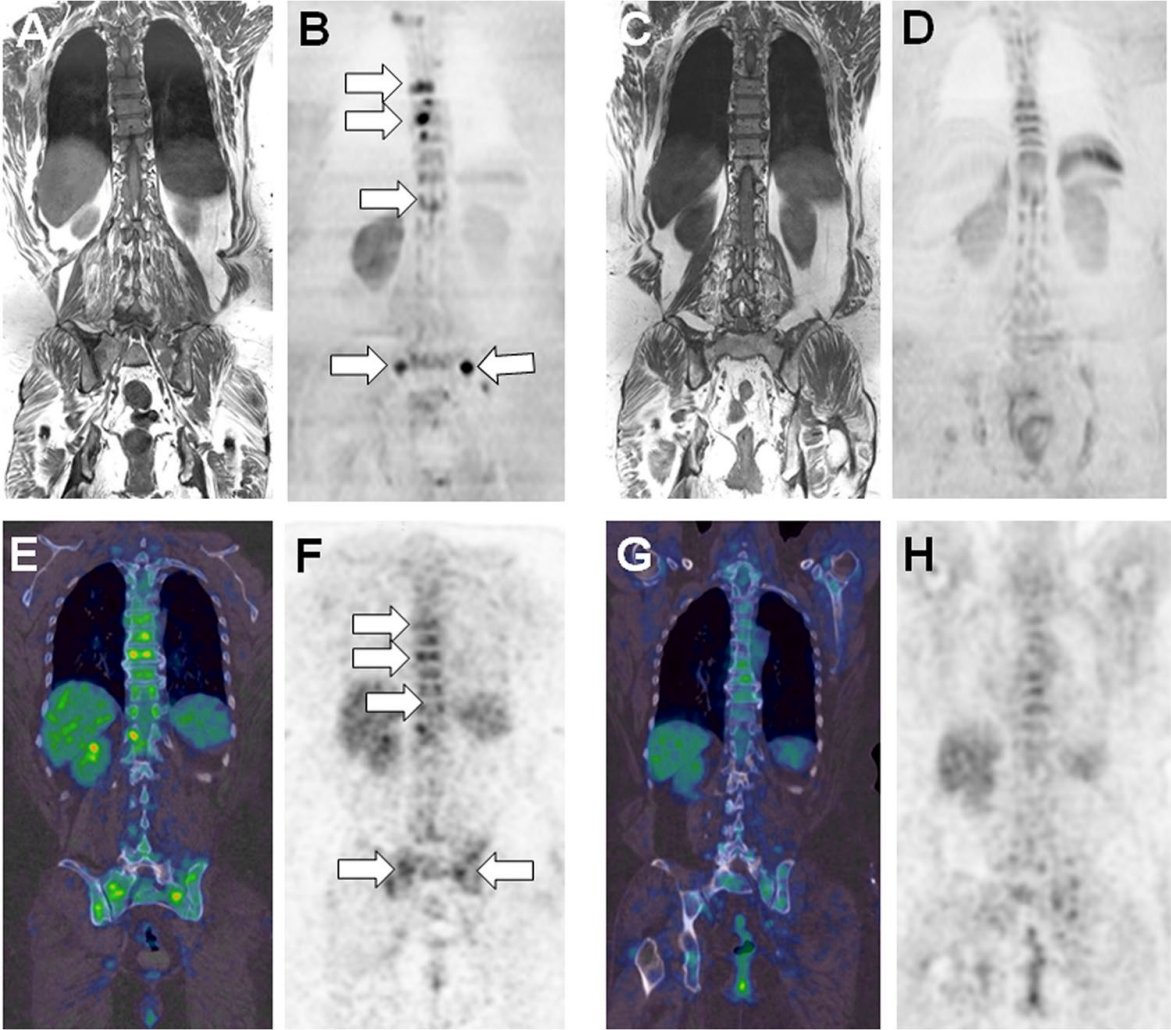
58 year-old woman with ND MM (IgA lambda, stage IIIA, ISS 2, with adverse cytogenetics (del 17p)). Comparison of WB-MRI and PET/CT findings at baseline and post-treatment (high dose cytotoxic treatment and ASCT). A–B Baseline MRI findings. Coronal T1 (A) and DWI (B = 1000 s/mm^2^) (B) MR images show active bone lesions within the spine and sacrum, barely seen on T1, much more evident on the DWI image (arrows in B). C–D Follow-up MRI. Coronal T1 (C) and DWI (B = 1000 s/mm^2^) (D) MR images show disappearance of all bone lesions. E–F Baseline PET/CT findings. Coronal fused PET/CT (E) and PET (F) show multiple foci with moderately increased [^18^F]FDG uptake (Deauville score 4) mainly involving the spine and sacrum (arrows in F) without evident lytic lesions or fractures on CT (not shown) (hottest bone lesion: T2 vertebral body, SUVmax 11.2; liver, SUVmax 4.2; mediastinal blood pool, SUVmax 2.8). G–H Following treatment, fused PET/CT (G) and PET (H) show disappearance of the focal PET uptake and reappearance of a homogeneous bone marrow uptake (Deauville score 3) suggesting a complete metabolic response (no residual disease at imaging) (T2 vertebral body, SUVmax 1.9; liver, SUVmax 4.0; mediastinal blood pool, SUVmax 2.7)

In an attempt of standardization and harmonization of FDG-PET/CT reporting and interpretation in daily practice and clinical trials, new visual descriptive criteria (Italian myeloma criteria for PET use, IMPeTUs) have been defined (Table 2) [109]. These criteria are a composite of visual assessment of FDG uptake using the 5-point Deauville score (initially designed for lymphoma) and typical MM-related features such as focal bone lesions, diffuse bone marrow uptake, fractures and para/extra-medullary lesions. The current IMWG criteria [9] stringently define the imaging response as disappearance of activity or residual activity below mediastinal blood pool (analogous to a Deauville score 1 or 2). Even though not yet incorporated in guidelines, a residual FDG uptake in focal lesions and bone marrow below liver background (analogous to a Deauville score 1–3) as reported by Zamagni et al. might replace the current IMWG response criteria in the future (Fig. 10).

### Limitations

[^18^F]FDG is a non-specific tracer that may accumulate in co-existing infectious or inflammatory disorders, degenerative joints or other benign conditions, hence leading to false-positive results. Both false negative or positive results can be obtained in relation to therapy for example soon after high dose corticosteroids, which transiently suppresses glucose metabolism, or after recent chemotherapy or growth factor injection that induce significant bone marrow stimulation. These confounders can be excluded by performing FDG-PET/CT studies > 1 month after these agents have been used. The most important cause of false-negative FDG-PET/CT results is associated with the lack of hexokinase in at least 10% of MM patients [110]. In this subgroup of baseline false-negative patients, FDG-PET/CT is not a suitable modality for monitoring response and detecting MRD. MRI and other PET tracers targeting alternative pathways should then be preferred [111].

### Alternative PET tracers

Several non-[^18^F]FDG tracers targeting other biological processes have successfully been evaluated in MM patients. Studies have been performed with lipid tracers such as [^11^C] or [^18^F]-choline or [^11^C]-acetate reflecting cell membrane synthesis and indirectly fatty acid synthesis, respectively [112]. Other studies have investigated amino acid tracers such as [^11^C]-methionine [113].

Recently, a PET tracer targeting the chemokine C-X-C motif receptor 4 (^68^Ga-CXCR4) has been developed driven by the knowledge that the CXCR4/SDF1 (stromal derived factor 1) axis may play an important role in various tumours and especially in MM. Studies in patient-derived MM cells have shown a strong correlation between CXCR4/SDF-1 activation and bone disease in MM [114]. It is known that SDF-1 engages CXCR4 on MM cells favouring their migration and extravasation to the bone marrow, an effect that is strengthened by the autocrine stimulation of plasma cells since MM cells themselves secrete SDF-1 [115]. The PET tracer targeting CXCR4 (^68^Ga-CXCR4) has been evaluated in MM patients and showed that CXCR4 expression frequently occurs in advanced MM and represents a negative prognostic factor [116]. This may provide an attractive strategy to select patients for CXCR4-targeted therapies either using anti-CXCR4 antibodies or for a theranostic approach with radionuclide therapy [117]. More recently, a CD38-targeted immune PET tracer, [^89^Zr]daratumumab, has successfully been synthetized and demonstrated tracer uptake at myeloma sites that were occult at routine MM imaging modalities [118]. However, these results remain very preliminary. The role of these tracers for response assessment and MRD detection is currently investigated.

### Comparisons of PET and MRI and value of the PET/MRI technology

Comparative studies have suggested that WB-MRI is more sensitive than PET/CT for the detection of bone marrow infiltration at diagnosis of MM thanks to its high spatial resolution and sensitivity to diffuse infiltration and due to the lack of FDG uptake in a significant (10–15%) proportion of MM patients which is most likely due to a low hexokinase-2 expression [87, 111, 119, 120].

Regarding response assessment, preliminary work suggests that FDG-PET/CT is more specific and allows early detection of relevant residual abnormalities (86% specificity for PET, 43% for WB-MRI; 75% sensitivity for both). In addition, FDG-PET/CT may become negative earlier after high dose therapy, while MRI may present residual marrow abnormalities [121, 122].

Current guidelines suggest that FDG-PET/CT should be considered the preferred imaging technique to monitor treatment response in MM. A prospective trial comparing AS-MRI (spine and pelvis) and PET/CT in treated MM patients suggested that PET/CT is superior to MRI for identifying MRD and providing prognostic information, showing improved PFS in patients showing normalization of the PET findings after 3 cycles of RVD and before maintenance therapy [104]. Another study comparing MRI and PET/CT found more false positive results of MRI after treatment, due to residual scar or necrotic tissue [123]. These studies were however limited by the lack of whole body coverage and use of DWI sequences which are known to improve the ability of MRI to distinguish viable from non-viable residual marrow abnormalities after treatment [99, 124].

A systematic review concluded that data on this topic are too heterogeneous, with biased accrual and lack of independent reference standard, which also precluded performing a meta-analysis [125]. Hence, it remains unclear which modality may be best suited for response assessment and the evaluation of both techniques using either side-by-side or on PET/MRI hybrid scanners should provide information on their respective effectiveness [126] (Figs. 5, 6, 10). PET/MRI indeed combines the two most effective imaging modalities in MM and allows a one-step exhaustive evaluation and comparison between both techniques at diagnosis, at the time of response assessment and MRD detection [99, 126].

The complementarity between WB-MRI/DWI and FDG-PET may be a key advantage of PET/MRI technology, especially to assess treatment response and MRD. Rasche et al. reported that WB-MRI/DWI identified a higher rate of lesions than FDG-PET/CT (21% vs. 6%), but not all PET-positive lesions were visible on DWI. This suggests that a combination of both methods might be necessary for optimal work-up and outcome prediction: patients with double positive or negative imaging findings after completion of the treatment had respectively a particularly poor and excellent PFS [28] (Fig. 10).

The same trial demonstrated the complementarity between imaging (both FDG-PET/CT and WB-MRI/DWI) and molecular techniques (flow cytometry) in defining the prognosis of patients [28]. Hence, for patients with CR, reaching double imaging and molecular MRD negativity is considered a predictive surrogate for patient outcome [127]. The prognostic value and therapeutic implications of positive imaging findings in patients with MRD bone marrow negativity deserve further evaluation. There is also a need for trials evaluating the individual value and potential complementarity of FDG-PET/CT and WB-MRI/DWI to monitor a sustained MRD negativity or its loss in high-risk MM patients [11].

## Conclusions

WB-MRI/DWI and PET/CT are the imaging methods of choice to assess response to treatment and to probe MRD in treated MM patients. Available imaging guidelines promote homogeneous use of the techniques and structured reporting amongst centres in daily routine and in clinical trials.

Regarding the clinical indications, WB-MRI and PET/CT should be obtained in advanced MM by the time of diagnosis before treatment, after completion of intensive treatment (ASCT) before maintenance and later on for systematic follow-up and by the time of suspected relapse. Further works should concentrate on the comparison of the value of both techniques by the time of response evaluation, detection of MRD and during follow-up under maintenance therapy. Their individual role in these indications and complementarity with modern molecular techniques deserve further assessment.

## Abbreviations

ASCT: Autologous stem cell transplantation
CR: Complete response
CT: Computed tomography
DCE: MRI Dynamic contrast enhanced magnetic resonance imaging
DWI: Diffusion weighted imaging
EMD: Extra medullary disease
[^18^F]FDG-PET: 2-deoxy-2-[^18^F] fluoro-D-glucose positron emission tomography
FF: Fat fraction
HR: High risk
IMiDs: Immunomodulatory drugs
IMWG: International Myeloma Working Group
ISS: International Staging System
MGUS: Monoclonal gammopathy of undetermined significance
MoAbs: Monoclonal antibodies
MIP: Maximal intensity projection
MM: Multiple myeloma
MPR: Multiplanar reformation
MRD: Minimal residual disease
MRI: Magnetic resonance imaging
MY-RADS: Myeloma Response Assessment and Diagnosis System
ND: Newly diagnosed
NGF: Next-generation flow
NGS: Next-generation sequencing
OS: Overall survival
PACS: Picture archiving and communication system
PET/CT: Positron emission tomography combined with computed tomography
PET/MRI: Positron emission tomography combined with MRI
PFS: Progression free survival
RAC: Response assessment categories
ROI: Region of interest
RSS: Radiographic skeletal survey
TIC: Time intensity curve
WB-CT: Whole body-computed tomography
WB-MRI: Whole body-magnetic resonance imaging

## References

[1] Kumar SK, Rajkumar V, Kyle RA (2017). Multiple myeloma Nature reviews Disease primers.

[2] Kyle RA, Gertz MA, Witzig TE (2003). Review of 1027 patients with newly diagnosed multiple myeloma. Mayo Clin Proc.

[3] Lecouvet FE, Malghem J, Michaux L (1999). Skeletal survey in advanced multiple myeloma: radiographic versus MR imaging survey. Br J Haematol.

[4] Dimopoulos M, Terpos E, Comenzo RL (2009). International myeloma working group consensus statement and guidelines regarding the current role of imaging techniques in the diagnosis and monitoring of multiple Myeloma. Leukemia.

[5] Baffour FI, Glazebrook KN, Kumar SK, Broski SM (2020). Role of imaging in multiple myeloma. Am J Hematol.

[6] Ocio EM, Richardson PG, Rajkumar SV (2014). New drugs and novel mechanisms of action in multiple myeloma in 2013: a report from the International Myeloma Working Group (IMWG). Leukemia.

[7] Horger M, Weisel K, Bares R (2011). Modern imaging techniques during therapy in patients with multiple myeloma. Acta radiologica (Stockholm, Sweden : 1987).

[8] Paiva B, van Dongen JJ, Orfao A (2015). New criteria for response assessment: role of minimal residual disease in multiple myeloma. Blood.

[9] Kumar S, Paiva B, Anderson KC (2016). International Myeloma Working Group consensus criteria for response and minimal residual disease assessment in multiple myeloma. Lancet Oncol.

[10] Latifoltojar A, Hall-Craggs M, Bainbridge A (2017). Whole-body MRI quantitative biomarkers are associated significantly with treatment response in patients with newly diagnosed symptomatic multiple myeloma following bortezomib induction. Eur Radiol.

[11] Zamagni E, Tacchetti P, Barbato S, Cavo M. Role of Imaging in the Evaluation of Minimal Residual Disease in Multiple Myeloma Patients. J Clin Med. 2020; 9(11). 10.3390/jcm9113519 PMC7692446 33142671

[12] Lecouvet FE, Vande Berg BC, Maldague BE (1997). Vertebral compression fractures in multiple myeloma. Part I. Distribution and appearance at MR imaging. Radiology.

[13] Kyle RA, Larson DR, Therneau TM (2018). Long-Term Follow-up of Monoclonal Gammopathy of Undetermined Significance. N Engl J Med.

[14] Rajkumar SV, Dimopoulos MA, Palumbo A (2014). International Myeloma Working Group updated criteria for the diagnosis of multiple myeloma. Lancet Oncol.

[15] Pawlyn C, Cairns D, Kaiser M (2020). The relative importance of factors predicting outcome for myeloma patients at different ages: results from 3894 patients in the Myeloma XI trial. Leukemia.

[16] Facon T, Dimopoulos MA, Meuleman N (2020). A simplified frailty scale predicts outcomes in transplant-ineligible patients with newly diagnosed multiple myeloma treated in the FIRST (MM-020) trial. Leukemia.

[17] Palumbo A, Bringhen S, Mateos MV (2015). Geriatric assessment predicts survival and toxicities in elderly myeloma patients: an International Myeloma Working Group report. Blood.

[18] Greipp PR, San Miguel J, Durie BG (2005). International staging system for multiple myeloma. J Clin Oncol.

[19] Avet-Loiseau H, Attal M, Campion L (2012). Long-term analysis of the IFM 99 trials for myeloma: cytogenetic abnormalities [t(4;14), del(17p), 1q gains] play a major role in defining long-term survival. J Clin Oncol.

[20] Hebraud B, Leleu X, Lauwers-Cances V (2014). Deletion of the 1p32 region is a major independent prognostic factor in young patients with myeloma: the IFM experience on 1195 patients. Leukemia.

[21] Palumbo A, Avet-Loiseau H, Oliva S (2015). Revised International Staging System for Multiple Myeloma: A Report From International Myeloma Working Group. J Clin Oncol.

[22] Gay F, Larocca A, Wijermans P (2011). Complete response correlates with long-term progression-free and overall survival in elderly myeloma treated with novel agents: analysis of 1175 patients. Blood.

[23] Lahuerta JJ, Paiva B, Vidriales MB (2017). Depth of Response in Multiple Myeloma: A Pooled Analysis of Three PETHEMA/GEM Clinical Trials. J Clin Oncol.

[24] Flores-Montero J, Sanoja-Flores L, Paiva B (2017). Next Generation Flow for highly sensitive and standardized detection of minimal residual disease in multiple myeloma. Leukemia.

[25] Faham M, Zheng J, Moorhead M (2012). Deep-sequencing approach for minimal residual disease detection in acute lymphoblastic leukemia. Blood.

[26] Kostopoulos IV, Ntanasis-Stathopoulos I, Gavriatopoulou M, Tsitsilonis OE, Terpos E (2020). Minimal Residual Disease in Multiple Myeloma: Current Landscape and Future Applications With Immunotherapeutic Approaches. Front Oncol.

[27] Paiva B, Puig N, Cedena MT (2020). Measurable Residual Disease by Next-Generation Flow Cytometry in Multiple Myeloma. J Clin Oncol.

[28] Rasche L, Alapat D, Kumar M (2019). Combination of flow cytometry and functional imaging for monitoring of residual disease in myeloma. Leukemia.

[29] Landgren O, Devlin S, Boulad M, Mailankody S (2016). Role of MRD status in relation to clinical outcomes in newly diagnosed multiple myeloma patients: a meta-analysis. Bone Marrow Transplant.

[30] Munshi NC, Avet-Loiseau H, Rawstron AC (2017). Association of Minimal Residual Disease With Superior Survival Outcomes in Patients With Multiple Myeloma: A Meta-analysis. JAMA Oncol.

[31] Perrot A, Lauwers-Cances V, Corre J (2018). Minimal residual disease negativity using deep sequencing is a major prognostic factor in multiple myeloma. Blood.

[32] Li H, Li F, Zhou X (2019). Achieving minimal residual disease-negative by multiparameter flow cytometry may ameliorate a poor prognosis in MM patients with high-risk cytogenetics: a retrospective single-center analysis. Ann Hematol.

[33] Durie BG, Salmon SE (1975). A clinical staging system for multiple myeloma. Correlation of measured myeloma cell mass with presenting clinical features, response to treatment, and survival. Cancer.

[34] Terpos E, Dimopoulos MA, Moulopoulos LA (2016). The Role of Imaging in the Treatment of Patients With Multiple Myeloma in 2016. Am Soc Clin Oncol Educ Book.

[35] Zamagni E, Cavo M, Fakhri B, Vij R, Roodman D (2018). Bones in Multiple Myeloma: Imaging and Therapy. Am Soc Clin Oncol Educ Book.

[36] Durie BG (2006). The role of anatomic and functional staging in myeloma: description of Durie/Salmon plus staging system. European journal of cancer (Oxford, England : 1990).

[37] Hillengass J, Usmani S, Rajkumar SV (2019). International myeloma working group consensus recommendations on imaging in monoclonal plasma cell disorders. Lancet Oncol.

[38] Princewill K, Kyere S, Awan O, Mulligan M (2013). Multiple myeloma lesion detection with whole body CT versus radiographic skeletal survey. Cancer Invest.

[39] Wolf MB, Murray F, Kilk K (2014). Sensitivity of whole-body CT and MRI versus projection radiography in the detection of osteolyses in patients with monoclonal plasma cell disease. Eur J Radiol.

[40] Hillengass J, Moulopoulos LA, Delorme S (2017). Whole-body computed tomography versus conventional skeletal survey in patients with multiple myeloma: a study of the International Myeloma Working Group. Blood Cancer J.

[41] Barwick T, Bretsztajn L, Wallitt K, Amiras D, Rockall A, Messiou C (2019). Imaging in myeloma with focus on advanced imaging techniques. Br J Radiol.

[42] Zamagni E, Tacchetti P, Cavo M (2019). Imaging in multiple myeloma: How? When?. Blood.

[43] Moreau P, San Miguel J, Sonneveld P (2017). Multiple myeloma: ESMO Clinical Practice Guidelines for diagnosis, treatment and follow-up. Annals of oncology : official journal of the European Society for Medical Oncology.

[44] Terpos E, Kleber M, Engelhardt M (2015). European Myeloma Network guidelines for the management of multiple myeloma-related complications. Haematologica.

[45] Horger M, Fritz J, Thaiss WM (2018). Comparison of qualitative and quantitative CT and MRI parameters for monitoring of longitudinal spine involvement in patients with multiple myeloma. Skeletal Radiol.

[46] Schulze M, Weisel K, Grandjean C (2014). Increasing bone sclerosis during bortezomib therapy in multiple myeloma patients: results of a reduced-dose whole-body MDCT study. AJR Am J Roentgenol.

[47] Reinert CP, Krieg EM, Bosmuller H, Horger M. Mid-term response assessment in multiple myeloma using a texture analysis approach on dual energy-CT-derived bone marrow images - A proof of principle study. Eur J Radiol. 2020; 131:109214. 10.1016/j.ejrad.2020.109214 32835853

[48] National Institute for Health and Care Excellence NICE. Myeloma: Diagnosis and management. Available via https://www.nice.org.uk/guidance/ng35/chapter/Recommendations. 2016. 32045178

[49] Lecouvet FE, Vande Berg BC, Malghem J, Maldague BE (2001). Magnetic resonance and computed tomography imaging in multiple myeloma. Semin Musculoskelet Radiol.

[50] Lecouvet FE, Whole-Body MR (2016). Imaging: Musculoskeletal Applications. Radiology.

[51] Larbi A, Omoumi P, Pasoglou V (2019). Whole-body MRI to assess bone involvement in prostate cancer and multiple myeloma: comparison of the diagnostic accuracies of the T1, short tau inversion recovery (STIR), and high b-values diffusion-weighted imaging (DWI) sequences. Eur Radiol.

[52] Lecouvet FE, Vande Berg BC, Michaux L (1998). Stage III multiple myeloma: clinical and prognostic value of spinal bone marrow MR imaging. Radiology.

[53] Larbi A, Omoumi P, Pasoglou V (2019). Comparison of bone lesion distribution between prostate cancer and multiple myeloma with whole-body MRI. Diagn Interv Imaging.

[54] Lecouvet FE, Simon M, Tombal B, Jamart J, Vande Berg BC, Simoni P (2010). Whole-body MRI (WB-MRI) versus axial skeleton MRI (AS-MRI) to detect and measure bone metastases in prostate cancer (PCa). Eur Radiol.

[55] Bauerle T, Hillengass J, Fechtner K (2009). Multiple myeloma and monoclonal gammopathy of undetermined significance: importance of whole-body versus spinal MR imaging. Radiology.

[56] Lecouvet FE, Pasoglou V, Van Nieuwenhove S (2020). Shortening the acquisition time of whole-body MRI: 3D T1 gradient echo Dixon vs fast spin echo for metastatic screening in prostate cancer. Eur Radiol.

[57] Pasoglou V, Michoux N, Peeters F (2015). Whole-body 3D T1-weighted MR imaging in patients with prostate cancer: feasibility and evaluation in screening for metastatic disease. Radiology.

[58] Bray TJP, Singh S, Latifoltojar A (2017). Diagnostic utility of whole body Dixon MRI in multiple myeloma: A multi-reader study. PLoS One.

[59] Maeder Y, Dunet V, Richard R, Becce F, Omoumi P (2018). Bone Marrow Metastases: T2-weighted Dixon Spin-Echo Fat Images Can Replace T1-weighted Spin-Echo Images. Radiology.

[60] Danner A, Brumpt E, Alilet M, Tio G, Omoumi P, Aubry S (2019). Improved contrast for myeloma focal lesions with T2-weighted Dixon images compared to T1-weighted images. Diagn Interv Imaging.

[61] Van Nieuwenhove S, Van Damme J, Padhani AR, et al. Whole-body magnetic resonance imaging for prostate cancer assessment: Current status and future directions. J Magn Reson Imaging. 2020. 10.1002/jmri.27485 33382151

[62] Perez-Lopez R, Nava Rodrigues D, Figueiredo I (2018). Multiparametric Magnetic Resonance Imaging of Prostate Cancer Bone Disease: Correlation With Bone Biopsy Histological and Molecular Features. Invest Radiol.

[63] Hillengass J, Bauerle T, Bartl R (2011). Diffusion-weighted imaging for non-invasive and quantitative monitoring of bone marrow infiltration in patients with monoclonal plasma cell disease: a comparative study with histology. Br J Haematol.

[64] Giles SL, deSouza NM, Collins DJ (2015). Assessing myeloma bone disease with whole-body diffusion-weighted imaging: comparison with x-ray skeletal survey by region and relationship with laboratory estimates of disease burden. Clin Radiol.

[65] Messiou C, Giles S, Collins DJ (2012). Assessing response of myeloma bone disease with diffusion-weighted MRI. Br J Radiol.

[66] Giles SL, Messiou C, Collins DJ (2014). Whole-body diffusion-weighted MR imaging for assessment of treatment response in myeloma. Radiology.

[67] Chen J, Li C, Tian Y (2019). Comparison of Whole-Body DWI and (18)F-FDG PET/CT for Detecting Intramedullary and Extramedullary Lesions in Multiple Myeloma. AJR Am J Roentgenol.

[68] Subhawong TK, Jacobs MA, Fayad LM (2014). Diffusion-weighted MR imaging for characterizing musculoskeletal lesions. Radiographics.

[69] Koutoulidis V, Fontara S, Terpos E (2017). Quantitative Diffusion-weighted Imaging of the Bone Marrow: An Adjunct Tool for the Diagnosis of a Diffuse MR Imaging Pattern in Patients with Multiple Myeloma. Radiology.

[70] Lecouvet FE, Vande Berg BC, Malghem J, Omoumi P, Simoni P (2009). Diffusion-weighted MR imaging: adjunct or alternative to T1-weighted MR imaging for prostate carcinoma bone metastases?. Radiology.

[71] Messiou C, Hillengass J, Delorme S (2019). Guidelines for Acquisition, Interpretation, and Reporting of Whole-Body MRI in Myeloma: Myeloma Response Assessment and Diagnosis System (MY-RADS). Radiology.

[72] Moulopoulos LA, Varma DG, Dimopoulos MA (1992). Multiple myeloma: spinal MR imaging in patients with untreated newly diagnosed disease. Radiology.

[73] Hillengass J, Ayyaz S, Kilk K (2012). Changes in magnetic resonance imaging before and after autologous stem cell transplantation correlate with response and survival in multiple myeloma. Haematologica.

[74] Lecouvet FE, Larbi A, Pasoglou V (2013). MRI for response assessment in metastatic bone disease. Eur Radiol.

[75] Baur-Melnyk A, Buhmann S, Durr HR, Reiser M (2005). Role of MRI for the diagnosis and prognosis of multiple myeloma. Eur J Radiol.

[76] Merz M, Hielscher T, Mai EK (2019). Cystic transformation of focal lesions after therapy is associated with remission but adverse outcome in myeloma. Blood Cancer J.

[77] Latifoltojar A, Hall-Craggs M, Rabin N (2017). Whole body magnetic resonance imaging in newly diagnosed multiple myeloma: early changes in lesional signal fat fraction predict disease response. Br J Haematol.

[78] Takasu M, Kondo S, Akiyama Y (2020). Assessment of early treatment response on MRI in multiple myeloma: Comparative study of whole-body diffusion-weighted and lumbar spinal MRI. PLoS One.

[79] Croft J, Riddell A, Koh DM (2020). Inter-observer agreement of baseline whole body MRI in multiple myeloma. Cancer Imaging.

[80] Almeida SD, Santinha J, Oliveira FPM (2020). Quantification of tumor burden in multiple myeloma by atlas-based semi-automatic segmentation of WB-DWI. Cancer Imaging.

[81] Blackledge MD, Tunariu N, Orton MR (2016). Inter- and Intra-Observer Repeatability of Quantitative Whole-Body, Diffusion-Weighted Imaging (WBDWI) in Metastatic Bone Disease. PLoS One.

[82] Durie BG, Harousseau JL, Miguel JS (2006). International uniform response criteria for multiple myeloma. Leukemia.

[83] Wu C, Huang J, Xu WB (2018). Discriminating Depth of Response to Therapy in Multiple Myeloma Using Whole-body Diffusion-weighted MRI with Apparent Diffusion Coefficient: Preliminary Results From a Single-center Study. Acad Radiol.

[84] Short KD, Rajkumar SV, Larson D (2011). Incidence of extramedullary disease in patients with multiple myeloma in the era of novel therapy, and the activity of pomalidomide on extramedullary myeloma. Leukemia.

[85] Lecouvet FE, Dechambre S, Malghem J, Ferrant A, Vande Berg BC, Maldague B (2001). Bone marrow transplantation in patients with multiple myeloma: prognostic significance of MR imaging. AJR Am J Roentgenol.

[86] Hillengass J, Merz M, Delorme S (2018). Minimal residual disease in multiple myeloma: use of magnetic resonance imaging. Semin Hematol.

[87] Pawlyn C, Fowkes L, Otero S (2016). Whole-body diffusion-weighted MRI: a new gold standard for assessing disease burden in patients with multiple myeloma?. Leukemia.

[88] Azad GK, Taylor BP, Green A (2019). Prediction of therapy response in bone-predominant metastatic breast cancer: comparison of [(18)F] fluorodeoxyglucose and [(18)F]-fluoride PET/CT with whole-body MRI with diffusion-weighted imaging. Eur J Nucl Med Mol Imaging.

[89] Cuenod CA, Laredo JD, Chevret S (1996). Acute vertebral collapse due to osteoporosis or malignancy: appearance on unenhanced and gadolinium-enhanced MR images. Radiology.

[90] Moulopoulos LA, Yoshimitsu K, Johnston DA, Leeds NE, Libshitz HI (1996). MR prediction of benign and malignant vertebral compression fractures. J Magn Reson Imaging.

[91] Lecouvet FE, Vande Berg BC, Michaux L, Jamart J, Maldague BE, Malghem J (1998). Development of vertebral fractures in patients with multiple myeloma: does MRI enable recognition of vertebrae that will collapse?. J Comput Assist Tomogr.

[92] Terpos E, Dimopoulos MA, Moulopoulos LA. The Role of Imaging in the Treatment of Patients With Multiple Myeloma in 2016. 2016(36):e407-e417 10.1200/EDBK_159074 27249748

[93] Dutoit JC, Vanderkerken MA, Verstraete KL (2013). Value of whole body MRI and dynamic contrast enhanced MRI in the diagnosis, follow-up and evaluation of disease activity and extent in multiple myeloma. Eur J Radiol.

[94] Koutoulidis V, Papanikolaou N, Moulopoulos LA (2018). Functional and molecular MRI of the bone marrow in multiple myeloma. Br J Radiol.

[95] Zechmann CM, Traine L, Meissner T (2012). Parametric histogram analysis of dynamic contrast-enhanced MRI in multiple myeloma: a technique to evaluate angiogenic response to therapy?. Acad Radiol.

[96] Dutoit JC, Claus E, Offner F, Noens L, Delanghe J, Verstraete KL (2016). Combined evaluation of conventional MRI, dynamic contrast-enhanced MRI and diffusion weighted imaging for response evaluation of patients with multiple myeloma. Eur J Radiol.

[97] Lin C, Luciani A, Belhadj K (2010). Multiple myeloma treatment response assessment with whole-body dynamic contrast-enhanced MR imaging. Radiology.

[98] Plathow C, Weber WA (2008). Tumor cell metabolism imaging. Journal of nuclear medicine : official publication, Society of Nuclear Medicine.

[99] Cavo M, Terpos E, Nanni C (2017). Role of (18)F-FDG PET/CT in the diagnosis and management of multiple myeloma and other plasma cell disorders: a consensus statement by the International Myeloma Working Group. Lancet Oncol.

[100] Bartel TB, Haessler J, Brown TL (2009). F18-fluorodeoxyglucose positron emission tomography in the context of other imaging techniques and prognostic factors in multiple myeloma. Blood.

[101] Davies FE, Rosenthal A, Rasche L (2018). Treatment to suppression of focal lesions on positron emission tomography-computed tomography is a therapeutic goal in newly diagnosed multiple myeloma. Haematologica.

[102] Zamagni E, Patriarca F, Nanni C (2011). Prognostic relevance of 18-F FDG PET/CT in newly diagnosed multiple myeloma patients treated with up-front autologous transplantation. Blood.

[103] Zamagni E, Nanni C, Mancuso K (2015). PET/CT Improves the Definition of Complete Response and Allows to Detect Otherwise Unidentifiable Skeletal Progression in Multiple Myeloma. Clin Cancer Res.

[104] Moreau P, Attal M, Caillot D (2017). Prospective Evaluation of Magnetic Resonance Imaging and [(18)F]Fluorodeoxyglucose Positron Emission Tomography-Computed Tomography at Diagnosis and Before Maintenance Therapy in Symptomatic Patients With Multiple Myeloma Included in the IFM/DFCI 2009 Trial: Results of the IMAJEM Study. J Clin Oncol.

[105] Zamagni E, Nanni C, Gay F (2019). MRD Evaluation By PET/CT According to Deauville Criteria Combined with Multiparameter Flow Cytometry in Newly Diagnosed Transplant Eligible Multiple Myeloma (MM) Patients Enrolled in the Phase II Randomized Forte Trial. Blood.

[106] Moreau P, Zweegman S, Perrot A (2019). Evaluation of the Prognostic Value of Positron Emission Tomography-Computed Tomography (PET-CT) at Diagnosis and Follow-up in Transplant-Eligible Newly Diagnosed Multiple Myeloma (TE NDMM) Patients Treated in the Phase 3 Cassiopeia Study: Results of the Cassiopet Companion Study. Blood.

[107] Zamagni E, Nanni C, Dozza L (2021). Standardization of (18)F-FDG-PET/CT According to Deauville Criteria for Metabolic Complete Response Definition in Newly Diagnosed Multiple Myeloma. J Clin Oncol.

[108] Meignan M, Itti E, Gallamini A, Haioun C (2009). Interim 18F-fluorodeoxyglucose positron emission tomography in diffuse large B-cell lymphoma: qualitative or quantitative interpretation–where do we stand?. Leuk Lymphoma.

[109] Nanni C. PET-FDG: Impetus. Cancers (Basel). 2020; 12(4). 10.3390/cancers12041030 PMC7226158 32331374

[110] Hillengass J, Landgren O (2013). Challenges and opportunities of novel imaging techniques in monoclonal plasma cell disorders: imaging "early myeloma". Leuk Lymphoma.

[111] Rasche L, Angtuaco E, McDonald JE (2017). Low expression of hexokinase-2 is associated with false-negative FDG-positron emission tomography in multiple myeloma. Blood.

[112] Nanni C, Zamagni E, Cavo M, et al. 11C-choline vs. 18F-FDG PET/CT in assessing bone involvement in patients with multiple myeloma. World J Surg Oncol. 2007; 5:68. 10.1186/1477-7819-5-68 PMC1913918 17584499

[113] Lapa C, Knop S, Schreder M (2016). 11C-Methionine-PET in Multiple Myeloma: Correlation with Clinical Parameters and Bone Marrow Involvement. Theranostics.

[114] Zannettino AC, Farrugia AN, Kortesidis A (2005). Elevated serum levels of stromal-derived factor-1alpha are associated with increased osteoclast activity and osteolytic bone disease in multiple myeloma patients. Cancer Res.

[115] Alsayed Y, Ngo H, Runnels J (2007). Mechanisms of regulation of CXCR4/SDF-1 (CXCL12)-dependent migration and homing in multiple myeloma. Blood.

[116] Lapa C, Schreder M, Schirbel A (2017). [(68)Ga]Pentixafor-PET/CT for imaging of chemokine receptor CXCR4 expression in multiple myeloma - Comparison to [(18)F]FDG and laboratory values. Theranostics.

[117] Herrmann K, Schottelius M, Lapa C (2016). First-in-Human Experience of CXCR4-Directed Endoradiotherapy with 177Lu- and 90Y-Labeled Pentixather in Advanced-Stage Multiple Myeloma with Extensive Intra- and Extramedullary Disease. Journal of nuclear medicine : official publication, Society of Nuclear Medicine.

[118] Ulaner GA, Sobol NB, O'Donoghue JA (2020). CD38-targeted Immuno-PET of Multiple Myeloma: From Xenograft Models to First-in-Human Imaging. Radiology.

[119] Kircher S, Stolzenburg A, Kortum KM (2019). Hexokinase-2 Expression in (11)C-Methionine-Positive, (18)F-FDG-Negative Multiple Myeloma. Journal of nuclear medicine : official publication, Society of Nuclear Medicine.

[120] Lecouvet FE, Boyadzhiev D, Collette L, et al. MRI versus (18)F-FDG-PET/CT for detecting bone marrow involvement in multiple myeloma: diagnostic performance and clinical relevance. Eur Radiol. 2019. 10.1007/s00330-019-06469-1 31844960

[121] Sachpekidis C, Mosebach J, Freitag MT (2015). Application of (18)F-FDG PET and diffusion weighted imaging (DWI) in multiple myeloma: comparison of functional imaging modalities. American journal of nuclear medicine and molecular imaging.

[122] Basha MAA, Hamed MAG, Refaat R (2018). Diagnostic performance of (18)F-FDG PET/CT and whole-body MRI before and early after treatment of multiple myeloma: a prospective comparative study. Jpn J Radiol.

[123] Derlin T, Peldschus K, Munster S (2013). Comparative diagnostic performance of (1)(8)F-FDG PET/CT versus whole-body MRI for determination of remission status in multiple myeloma after stem cell transplantation. Eur Radiol.

[124] Park HY, Kim KW, Yoon MA (2020). Role of whole-body MRI for treatment response assessment in multiple myeloma: comparison between clinical response and imaging response. Cancer Imaging.

[125] Gariani J, Westerland O, Natas S, Verma H, Cook G, Goh V (2018). Comparison of whole body magnetic resonance imaging (WBMRI) to whole body computed tomography (WBCT) or (18)F-fluorodeoxyglucose positron emission tomography/CT ((18)F-FDG PET/CT) in patients with myeloma: Systematic review of diagnostic performance. Crit Rev Oncol Hematol.

[126] Mule S, Reizine E, Blanc-Durand P, et al. Whole-Body Functional MRI and PET/MRI in Multiple Myeloma. Cancers (Basel). 2020; 12(11). 10.3390/cancers12113155 PMC7693006 33121132

[127] Jamet B, Zamagni E, Nanni C, et al. Functional Imaging for Therapeutic Assessment and Minimal Residual Disease Detection in Multiple Myeloma. Int J Mol Sci. 2020; 21(15). 10.3390/ijms21155406 PMC7432032 32751375

